# Periodontitis-Associated Transcriptional Signatures and Their Correspondence with Oral Mucosal Responses to Radiotherapy: A Cross-Cohort Analysis

**DOI:** 10.3390/ijms27219976

**Published:** 2026-11-07

**Authors:** Ziqiang Liu, Ronghan Huang, Feiyuan Xue, Xiaodan Feng, Shichun Gao, Zhendong Gong, Lin Liu, Runting Wang, Yanni Wu, Guozhu Xie, Lanfang Zhang

**Affiliations:** 1 Department of Radiation Oncology, Nanfang Hospital, Southern Medical University, No. 1838 North Guangzhou Avenue, Guangzhou 510515, China; 18137860058@163.com (Z.L.); hrh18470727937@163.com (R.H.); xuefeiyuan814@outlook.com (F.X.); fengf13026769110@163.com (X.F.); gaoshichun2024@163.com (S.G.); 2 Department of Stomatology, Nanfang Hospital, Southern Medical University, Guangzhou 510515, China; g410279488@163.com (Z.G.); llsp609409@163.com (L.L.); wrunting@163.com (R.W.); 3 Nanfang Hospital, Southern Medical University, Guangzhou 510515, China; yanniwusmu@126.com

**Keywords:** periodontitis, oral mucositis, radiotherapy, transcriptomics, gene expression signature, gene-set enrichment, cross-cohort analysis

## Abstract

The extent to which periodontitis-associated transcriptional patterns correspond to oral mucosal responses to radiotherapy remains unclear. We integrated three human transcriptomic cohorts to characterize this correspondence and its analytical stability using differential-expression, signature-score, and gene-set analyses. In GSE10334, a patient-blocked analysis of 247 gingival profiles identified 111 differentially expressed genes, of which 104 retained significance across sensitivity analyses. In the independent GSE173078 cohort, 95 of 97 mapped genes showed concordant effect directions (Spearman ρ = 0.824). Twenty-three genes met replication criteria in the fully adjusted model, compared with none in the batch-only model. The signature score was higher in periodontitis than in healthy gingiva (β = 1.66; 95% confidence interval, 0.40–2.93). In GSE103412, comprising eight patients with seven post-treatment observations, neither the 18 mapped replicated periodontal genes nor longitudinal signature scores met significance criteria. CAMERA detected enrichment of the periodontitis-upregulated set during and after radiotherapy with intergene correlation fixed at 0.01 (false discovery rate [FDR] = 0.00124 and 4.70 × 10^−6^), but not with data-estimated correlation (FDR = 0.360 and 0.177). Expression-matched random-set comparisons indicated signals exceeding the specified background without establishing disease specificity. Twenty-one overlapping Gene Ontology terms showed concordant cross-cohort enrichment under the original analytical specification. Cellular-proxy analyses suggested contributions from tissue composition or cellular state, while exploratory associations with mucositis severity were nonsignificant after correction. These findings identify partial, analytically dependent transcriptional correspondence without establishing that periodontitis increases susceptibility to radiation-induced mucositis or provides clinical predictive value. Prospective studies incorporating periodontal assessment and concurrent molecular and clinical measurements are needed.

## 1. Introduction

Oral mucositis is a frequent and clinically consequential toxicity of radiotherapy for head and neck cancer [1]. The resulting pain and mucosal injury can impair oral intake and increase requirements for analgesia, nutritional support, and other supportive care [2]. Severe mucositis remains common even with modern intensity-modulated radiotherapy, underscoring the need to better characterize its underlying tissue responses. Longitudinal profiling of human oral mucosa has identified treatment-associated changes in transcripts governing apoptosis, DNA-damage responses, and cell proliferation [3].

Periodontitis offers a complementary model of chronic inflammation and disrupted tissue homeostasis at the oral barrier. Integrated host-microbial studies have linked the disease to dysbiotic communities and altered host transcription, whereas gingival transcriptomic analyses have identified changes in immune signaling and related biological processes [4,5,6]. At single-cell resolution, periodontal inflammation reflects coordinated shifts across epithelial, stromal, and immune compartments, including inflammatory stromal states associated with increased leukocyte recruitment [7,8,9]. Periodontitis-associated signatures may therefore capture broader features of an inflamed oral tissue microenvironment that could correspond to responses induced by radiotherapy.

The extent to which these periodontal expression patterns correspond to longitudinal changes in irradiated oral mucosa remains unclear. This question can be examined at the levels of individual genes, aggregate sample scores, and biological processes. Establishing the degree of agreement at each level may help define which features of chronic periodontal inflammation are also represented in the mucosal response to treatment.

Publicly available human transcriptomic cohorts enable this question to be examined across complementary study designs. GSE10334 contains periodontitis-affected and clinically healthy gingival sites sampled within individuals, supporting discovery analyses that account for within-patient dependence [10]. GSE173078 is an independent RNA-sequencing cohort spanning periodontal health, gingivitis, and periodontitis, allowing assessment across cohorts and platforms [5]. GSE103412 comprises repeated oral mucosal samples collected before, during, and after radiotherapy, permitting within-patient evaluation of treatment-associated changes [3]. Together, these cohorts provide a framework for independent evaluation of a periodontal signature followed by its assessment in longitudinal radiotherapy samples.

We hypothesized that a periodontitis-associated transcriptional signature would show partial correspondence with longitudinal oral mucosal responses to radiotherapy, potentially differing across gene-, score-, and biological-process-level analyses. The study aimed to characterize the extent and analytical stability of this correspondence across three independent cohorts.

## 2. Results

### 2.1. Identification of a Periodontitis-Associated Transcriptional Signature

The GSE10334 discovery cohort comprised 247 gingival samples from 90 individuals, including 183 periodontitis-affected sites and 64 clinically healthy sites. The primary differential-expression model accounted for correlations among samples obtained from the same individual. Normalized expression distributions were broadly comparable across samples, whereas principal-component analysis showed partial separation by clinical status (Figure S1).

**Figure 1:**
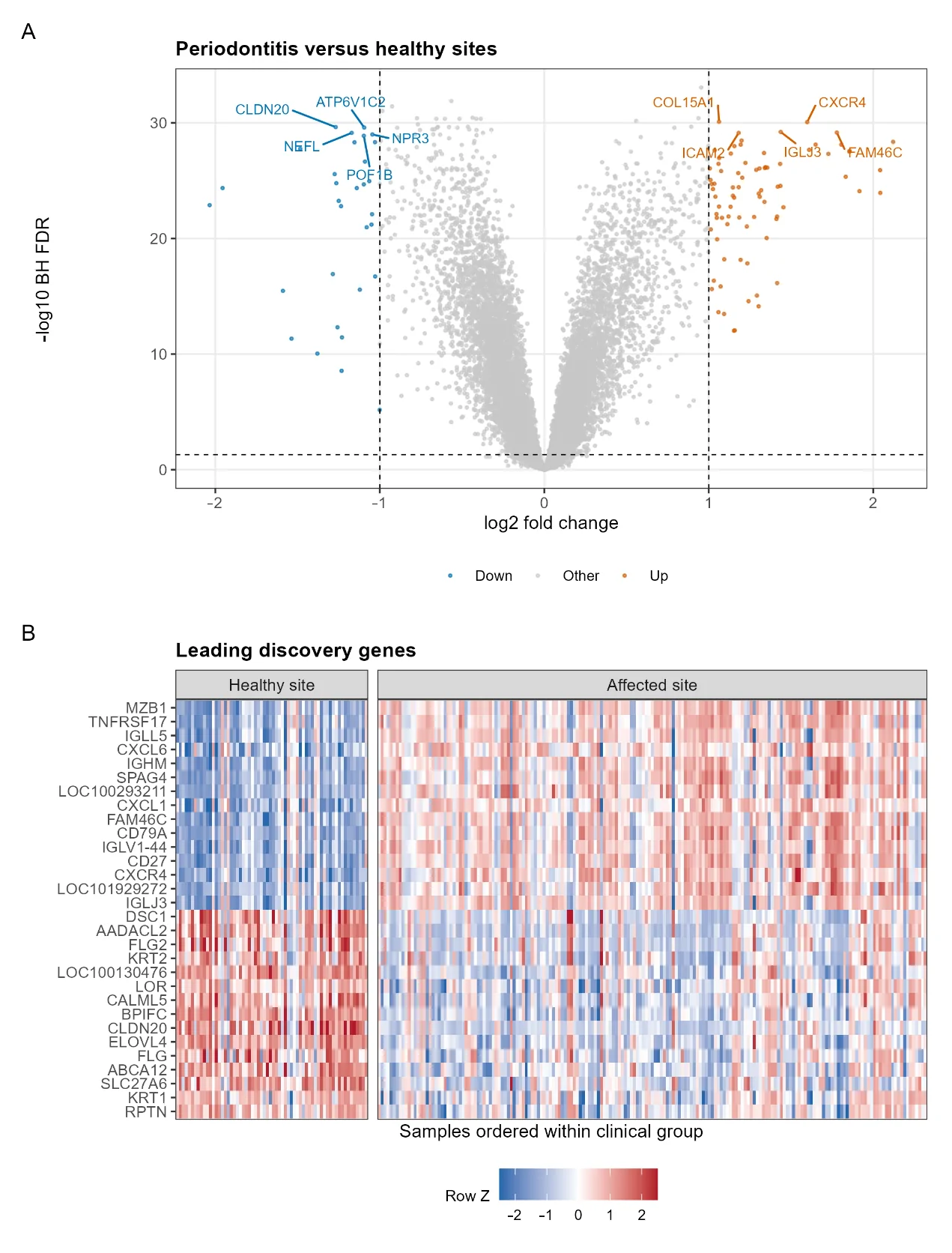
Identification of the periodontitis-associated transcriptional signature in GSE10334. (**A**) Volcano plot of differential expression between 183 periodontitis-affected and 64 clinically healthy gingival sites from 90 individuals, estimated using the patient-blocked model. Orange and blue points denote selected upregulated and downregulated genes, respectively, whereas gray points denote genes that did not meet both selection criteria. Dashed lines indicate |log2 fold change| > 1 and Benjamini–Hochberg false discovery rate (FDR) < 0.05. A total of 80 upregulated and 31 downregulated genes met this joint selection rule; up to five leading genes in each direction are labeled. (**B**) Heatmap of the 15 most upregulated and 15 most downregulated selected genes. Columns represent samples ordered by clinical group and patient, rows represent genes, and colors indicate row-wise z scores. The displayed color scale is truncated at −2.5 and 2.5. Quality-control plots are provided in Figure S1.

Using the combined criteria of |log_2_ fold change (FC)| > 1 and Benjamini–Hochberg false discovery rate (FDR) < 0.05, the primary analysis identified 111 differentially expressed genes, comprising 80 upregulated and 31 downregulated genes (Figure 1A). Upregulated genes included MZB1, TNFRSF17, IGLL5, FAM46C, CD79A, CD27, CXCR4, IL10RA, and CXCL1, whereas CLDN20, ATP6V1C2, NEFL, NPR3, POF1B, and SPAG17 were among the strongly downregulated genes. The expression heatmap of the 15 most upregulated and 15 most downregulated selected genes illustrated contrasting transcriptional patterns between affected and clinically healthy sites (Figure 1B).

Two sensitivity analyses assessed the dependence of these findings on sample inclusion and the handling of repeated measurements. Excluding three samples identified as extreme in the original principal-component analysis yielded 118 differentially expressed genes, whereas analysis of paired patient-level mean expression yielded 106. Of the 111 genes selected in the primary analysis, 104 met the differential-expression criteria in all three models. Genome-wide effect estimates were also closely correlated between the primary and sensitivity analyses (Pearson r = 0.997–0.999). These results supported the stability of the discovery signature within GSE10334 under the evaluated analytical alternatives.

### 2.2. Independent Evaluation Reveals Consistent Effect Directions but Model-Dependent Gene-Level Replication

The signature was evaluated in GSE173078, an independent RNA-sequencing cohort containing 12 healthy, 12 gingivitis, and 12 periodontitis samples. Normalized expression distributions were broadly comparable, and principal-component analysis showed pronounced transcriptomic structure across samples (Figure S2). The fully adjusted design incorporated phenotype, sequencing batch, age, and sex and was of full rank, with 30 residual degrees of freedom. Phenotype groups were evenly distributed across sequencing batches; however, the periodontitis group had a descriptively higher mean age and a lower proportion of women than the healthy group (Table 1A).

**Figure 2:**
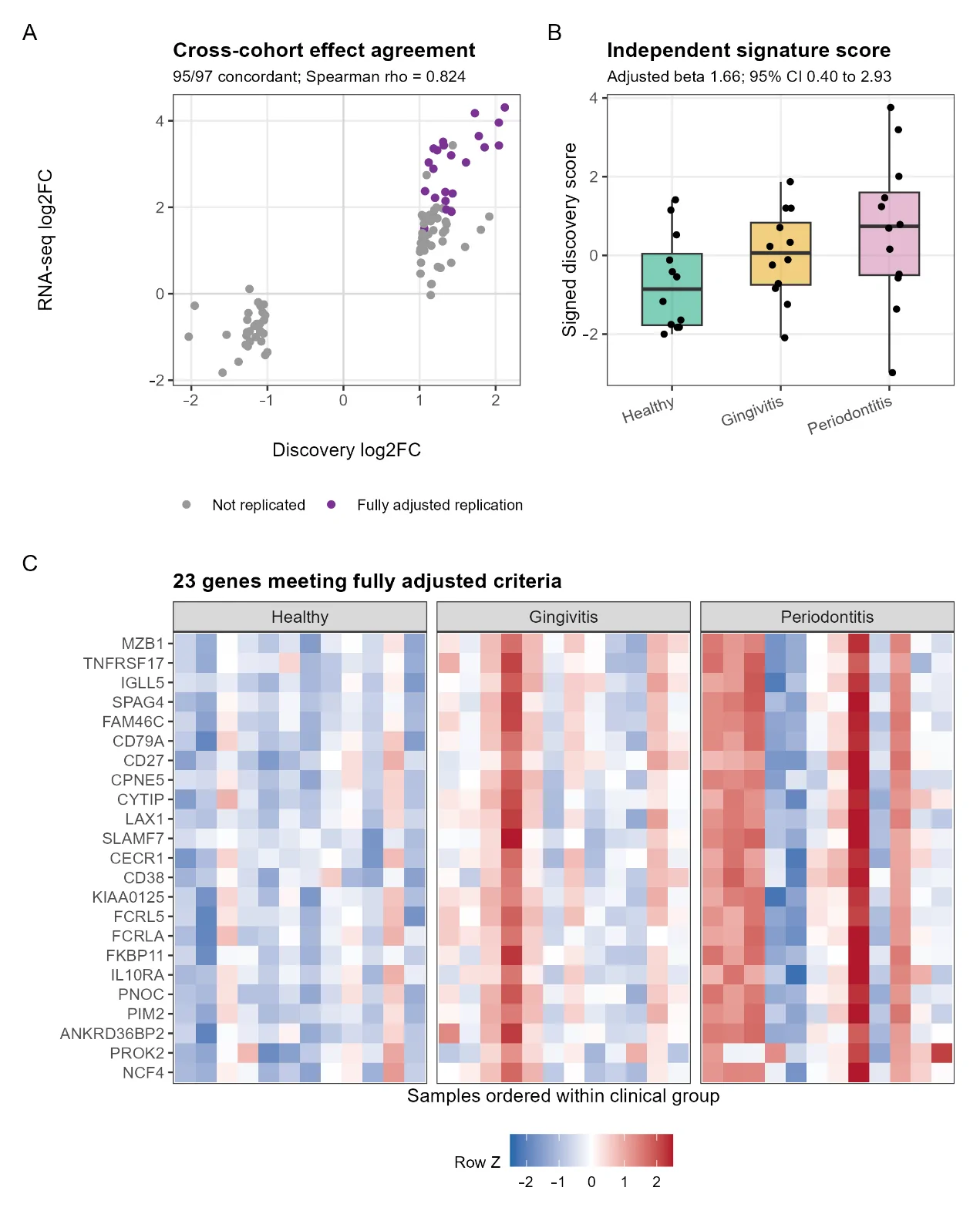
Independent evaluation of the periodontal transcriptional signature in GSE173078. (**A**) Concordance of log2 fold-change estimates for the 97 discovery genes represented in both periodontal cohorts. Purple points denote the 23 genes with concordant effects that met FDR < 0.05 and |log2 fold change| > 1 in the RNA-sequencing model adjusted for batch, age, and sex; gray points denote the remaining mapped genes. Ninety-five of the 97 genes showed concordant effect directions (Spearman ρ = 0.824). (**B**) Distribution of the signed discovery score, calculated from 97 mapped genes, across healthy gingiva, gingivitis, and periodontitis groups (*n* = 12 per group). Points represent individual samples; boxes indicate the median and interquartile range, and whiskers extend to 1.5 times the interquartile range. The adjusted periodontitis-versus-health coefficient was 1.66 (95% confidence interval [CI], 0.40–2.93; *p* = 0.0119). (**C**) Heatmap of the 23 genes meeting the fully adjusted replication criteria. Columns represent samples grouped by phenotype, rows represent genes, and colors indicate row-wise z scores, with the displayed scale truncated at ±2.5. Selection of these genes was conditional on the fully adjusted model; model and sample sensitivity analyses are presented in Figure S4.

Of the 111 discovery genes, 97 were represented in the RNA-sequencing dataset. Their effect estimates were strongly correlated with those in the discovery cohort (Spearman ρ = 0.824), and 95 of the 97 genes showed concordant directions of change (Figure 2A). In the fully adjusted periodontitis-versus-healthy comparison, 135 genes met the combined fold-change and FDR criteria, including 119 upregulated and 16 downregulated genes. Twenty-three discovery genes met the replication criteria in this model; all were upregulated (Figure 2A,C). Representative genes included MZB1 (log_2_FC = 4.31, FDR = 0.0218), TNFRSF17 (log_2_FC = 3.43, FDR = 0.0447), IGLL5 (log_2_FC = 3.96, FDR = 0.0279), CD79A (log_2_FC = 4.17, FDR = 0.0275), CD27 (log_2_FC = 3.04, FDR = 0.0284), FCRL5 (log_2_FC = 3.51, FDR = 0.0411), and IL10RA (log_2_FC = 1.77, FDR = 0.0495).

Gene-level significance varied substantially with covariate specification. The batch-only, batch-plus-age, batch-plus-sex, and fully adjusted models identified 4, 111, 1, and 135 differentially expressed genes, respectively (Figure S4A). The corresponding numbers of replicated discovery genes were 0, 22, 0, and 23. Although genome-wide effect estimates remained strongly correlated across models (Pearson r = 0.951–0.984), agreement in effect estimates did not translate into consistent threshold-based replication.

Leave-one-sample-out analyses further distinguished directional consistency from statistical stability. Across 36 refitted models, all 23 genes retained positive effect estimates, but the number meeting the replication criteria ranged from 0 to 23 per run. Individual genes met these criteria in 10–34 of the 36 runs; none did so in every run (Figure S4B). Exclusion of either AC022 or AC039 resulted in none of the original 23 genes meeting the replication thresholds. These analyses retained the original filtering universe and normalization factors while re-estimating dispersion and model coefficients.

Effect and precision diagnostics did not support a uniform reduction in uncertainty as the explanation for the increase in significant genes after covariate adjustment. Among the 23 genes, the median edgeR log_2_FC increased from 2.69 in the batch-only model to 3.04 in the fully adjusted model, whereas the median ratio of quasi-likelihood posterior dispersions was approximately 0.975. In a separate voom–limma analysis, the median fully adjusted-to-batch-only standard-error ratio was approximately 1.04 (Figure S9). Thus, the independent cohort supported concordant effect directions, while gene-level replication remained sensitive to both model specification and sample inclusion.

### 2.3. Aggregate Periodontal Scores Remain Associated with Periodontitis After Covariate Adjustment

The primary signed discovery score was defined as the mean standardized expression of mapped upregulated genes minus that of mapped downregulated genes. In GSE173078, this score incorporated 97 genes: 67 upregulated and 30 downregulated. After adjustment for batch, age, and sex, the periodontitis group had a higher score than the healthy group (β = 1.66, 95% confidence interval [CI] 0.40–2.93, *p* = 0.0119; Figure 2B). The gingivitis-versus-healthy contrast was not significant (*p* = 0.275).

The periodontitis association was also detected in the batch-only model (β = 1.34, 95% CI 0.12–2.57, *p* = 0.0329; Table 1B). Across these two specifications, the aggregate score association was therefore more consistent than threshold-based replication of individual genes.

A separate, equal-gene-weight score was used for expression-matched random-signature testing. This implementation averaged standardized expression after assigning each gene its discovery direction and therefore differed in scale and weighting from the primary upregulated-minus-downregulated score. Harmonization of the identifier C16ORF54/C16orf54 increased the mapped gene count in this implementation from 96 to 97. The correction had little effect on the adjusted periodontitis coefficient: the estimate changed from 0.8298 to 0.8312, with the corrected score yielding a 95% CI of 0.1933–1.4691 and *p* = 0.0124.

The absolute adjusted effect of the corrected 97-gene score exceeded those of all 1000 random signatures matched for gene-set size and mean-expression decile, yielding a two-sided empirical *p* value of 0.000999. This comparison supported an association exceeding that observed for the specified expression-matched background, but did not test specificity relative to other inflammatory diseases.

### 2.4. Radiotherapy-Associated Transcriptional Changes in Oral Mucosa

GSE103412 contained 32 oral-mucosa profiles: nine healthy-control profiles, eight patient baseline profiles, eight during-radiotherapy profiles, and seven post-radiotherapy profiles. Longitudinal analyses used the 23 patient profiles, providing eight baseline–during-treatment pairs and seven baseline–post-treatment pairs. Healthy controls were included in descriptive quality-control analyses but were not used as baseline observations in the patient-level models. Normalized expression distributions were broadly comparable, and principal-component analysis showed overlap among patient samples across time points, with greater dispersion among healthy controls (Figure S3).

**Figure 3:**
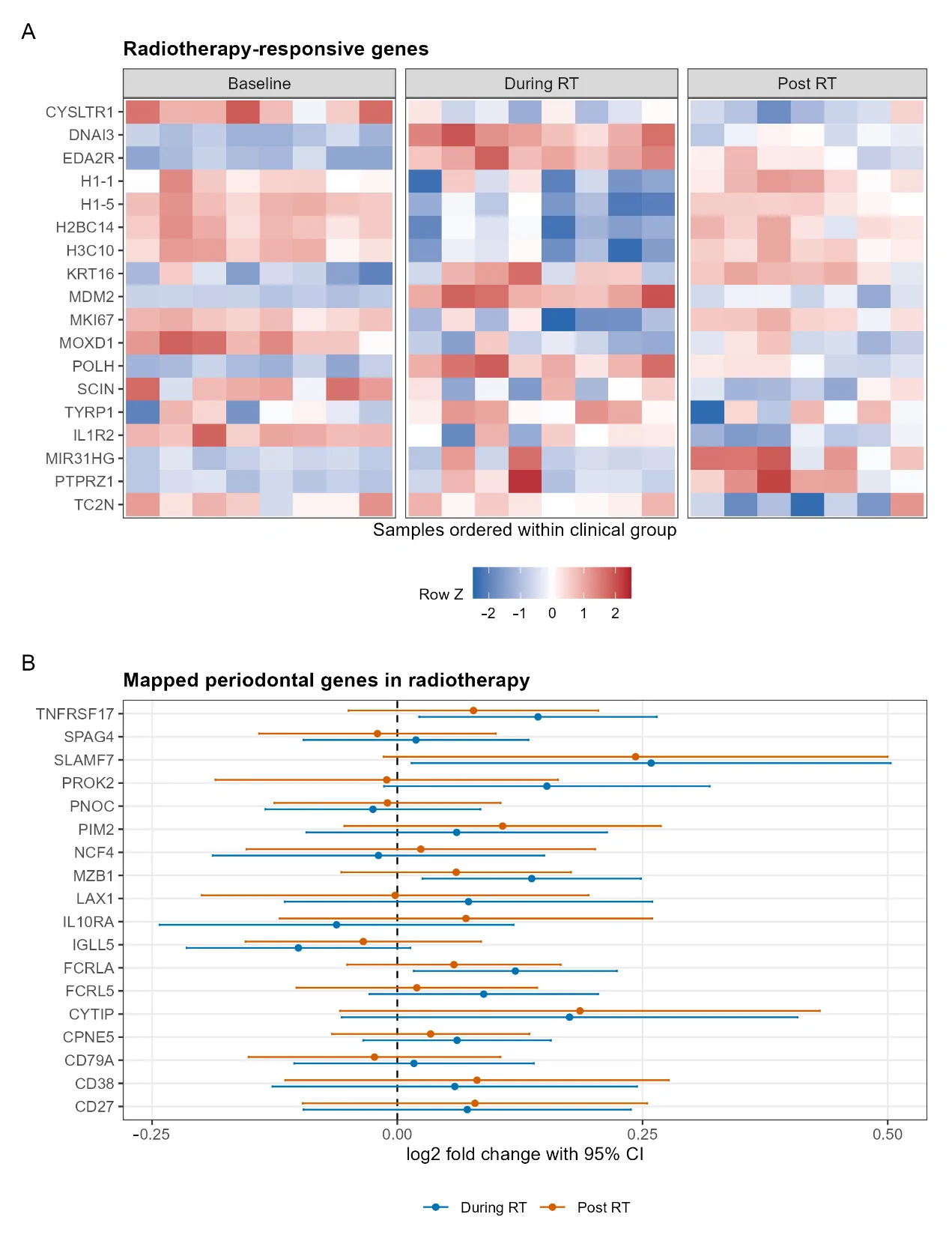
Longitudinal oral-mucosal expression changes and periodontal gene-level correspondence in GSE103412. (**A**) Heatmap of the 18 genes meeting FDR < 0.05 and |log2 fold change| > 1 in at least one comparison with baseline. Columns represent patient samples ordered by time point and patient, rows represent genes, and colors indicate row-wise z scores, with the displayed scale truncated at ±2.5. (**B**) Log2 fold-change estimates and 95% CIs for the 18 of 23 replicated periodontal genes represented in GSE103412. Points indicate model estimates, and horizontal lines indicate 95% CIs; blue and orange denote during-radiotherapy and post-radiotherapy comparisons with baseline, respectively. The dashed vertical line indicates no change. None of these periodontal genes reached gene-level FDR < 0.05 in either comparison. Panels (**A**,**B**) depict different gene sets despite both containing 18 genes. Models included fixed patient effects and used 23 profiles from eight patients: eight baseline, eight during-treatment, and seven post-treatment samples. Corresponding volcano plots are provided in Figure S5.

In patient-adjusted comparisons with baseline, 14 genes met the combined criteria of |log_2_FC| > 1 and FDR < 0.05 during radiotherapy, including six upregulated and eight downregulated genes (Figure S5A). Upregulated genes included MDM2, DNAI3, POLH, EDA2R, KRT16, and TYRP1; downregulated genes included H1-5, H3C10, H2BC14, MKI67, CYSLTR1, and SCIN. Seven genes met these criteria after radiotherapy: KRT16, MIR31HG, and PTPRZ1 were upregulated, whereas IL1R2, CYSLTR1, TC2N, and SCIN were downregulated (Figure S5B). The union of the two comparisons comprised 18 genes with distinct temporal expression patterns (Figure 3A).

Without imposing a fold-change threshold, the unified patient-adjusted model identified 755 genes at FDR < 0.05 during radiotherapy versus baseline, 148 after radiotherapy versus baseline, and 348 after versus during radiotherapy. Of these, 14, 7, and 7 genes, respectively, also exceeded |log_2_FC| > 1. Thus, most statistically significant transcriptional differences were smaller than a twofold change.

Of the 23 periodontal genes replicated in the fully adjusted RNA-sequencing analysis, 18 were represented in the radiotherapy cohort. None reached gene-level FDR < 0.05 in either comparison with baseline, and their effect estimates had wide confidence intervals (Figure 3B). These mapped periodontal genes were distinct from the 18 radiotherapy-responsive genes displayed in Figure 3A.

### 2.5. Periodontal Gene-Set Enrichment Depends on Correlation Assumptions and Is Not Accompanied by Significant Patient-Level Score Changes

**Figure 4:**
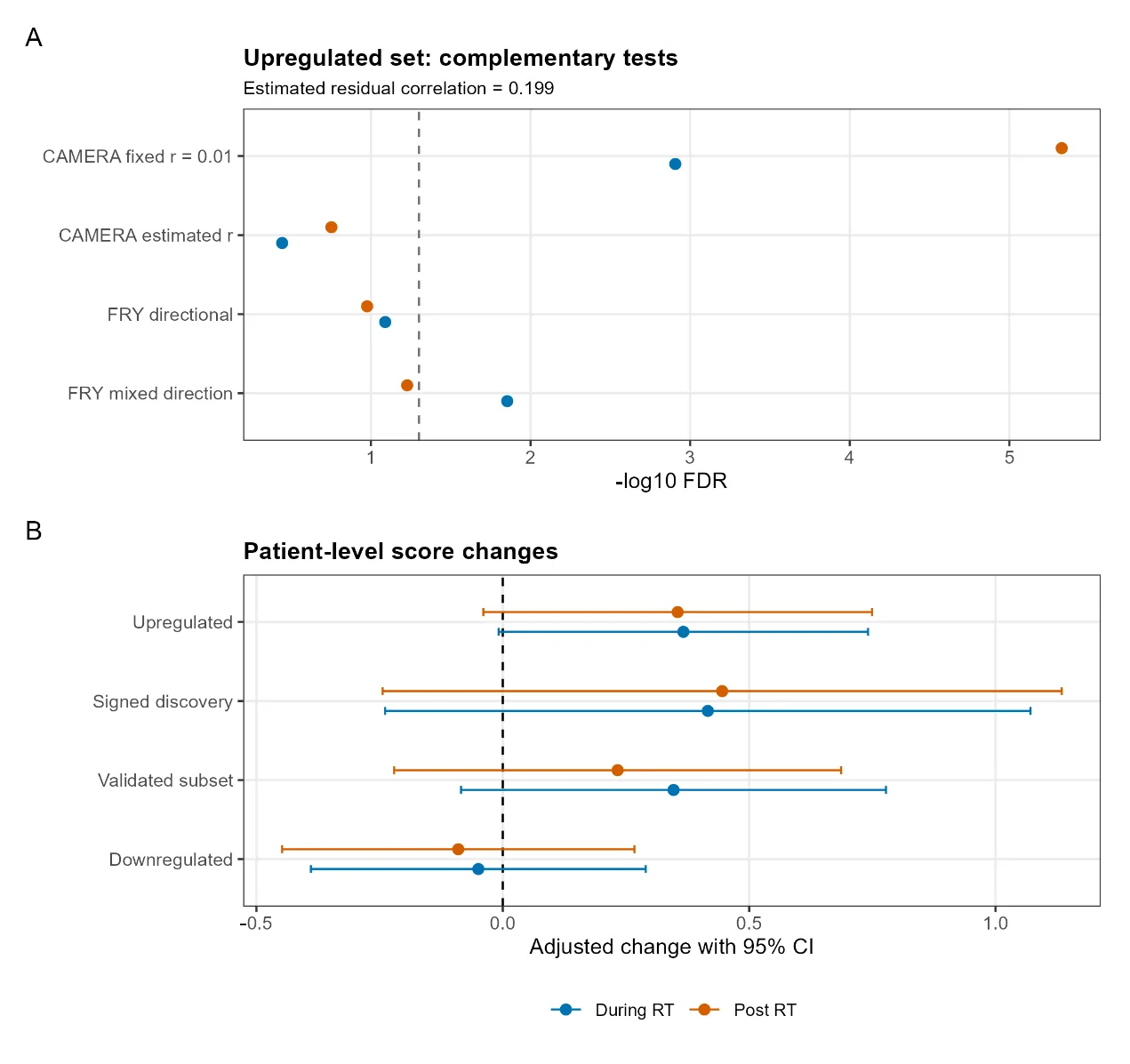
Complementary gene-set tests and longitudinal periodontal signature scores in GSE103412. (**A**) Enrichment of the 63-gene periodontitis-upregulated set evaluated using CAMERA with fixed intergene correlation of 0.01, CAMERA with data-estimated correlation of approximately 0.199, and directional and mixed-direction FRY tests. Points indicate −log10 FDR for comparisons during and after radiotherapy with baseline; the dashed line marks FDR = 0.05. FDR adjustment was performed within the respective three-set testing family at each time point. Mixed-direction FRY significance does not imply a concordant increase in expression. (**B**) Adjusted changes in the upregulated component (63 genes), downregulated component (28 genes), signed discovery score (91 mapped genes), and replicated-gene score (18 mapped genes). Points indicate estimates from fixed-patient models, and horizontal lines indicate 95% CIs; the dashed line marks no change. Gene standardization was based on the same 23 patient profiles used in the primary score analysis. Blue and orange indicate during-radiotherapy and post-radiotherapy comparisons, respectively. All score-change CIs include zero. The enrichment results depend on the correlation specification and do not establish consistent elevation of the complete periodontal signature across patients.

The radiotherapy dataset contained 91 discovery-signature genes, comprising 63 periodontitis-upregulated and 28 periodontitis-downregulated genes. Under the original CAMERA specification, with intergene correlation fixed at 0.01, the upregulated subset was enriched during radiotherapy (FDR = 0.00124) and after radiotherapy (FDR = 4.70 × 10^−6^; Figure 4A). The complete 91-gene set also met the significance threshold at both time points (FDR = 0.0295 and 0.00181, respectively), whereas the downregulated subset did not.

These findings were sensitive to the treatment of intergene correlation. The estimated residual correlation for the upregulated subset was approximately 0.199. When CAMERA used the data-estimated correlation, the upregulated subset no longer met FDR < 0.05 during or after radiotherapy (FDR = 0.360 and 0.177, respectively). The complete signature likewise did not meet this threshold. Complementary FRY directional tests were nonsignificant for the upregulated subset at both time points (FDR = 0.0814 and 0.1057). The FRY mixed-direction test was significant during radiotherapy (FDR = 0.0140) but not after treatment (FDR = 0.0593). Mixed-direction tests also detected changes in the downregulated subset and complete signature at both time points; these results indicate expression changes within the sets without establishing a concordant shift in the periodontal direction.

Patient-level scores provided a separate assessment of longitudinal change (Figure 4B). The signed discovery score increased by an estimated 0.42 during radiotherapy (95% CI −0.24 to 1.07, *p* = 0.193) and 0.45 after radiotherapy (95% CI −0.24 to 1.13, *p* = 0.186). For the replicated-gene subset score, the corresponding estimates were 0.35 (95% CI −0.08 to 0.78, *p* = 0.106) and 0.23 (95% CI −0.22 to 0.69, *p* = 0.287).

Separating the upregulated and downregulated components did not identify a significant patient-level change. The upregulated-component estimates were 0.366 during treatment (95% CI −0.008 to 0.741, *p* = 0.0546) and 0.355 after treatment (95% CI −0.040 to 0.749, *p* = 0.0739). The downregulated-component estimates were −0.050 and −0.090, respectively, with both confidence intervals including zero. None of the eight component-by-time comparisons met FDR < 0.05.

**Figure 5:**
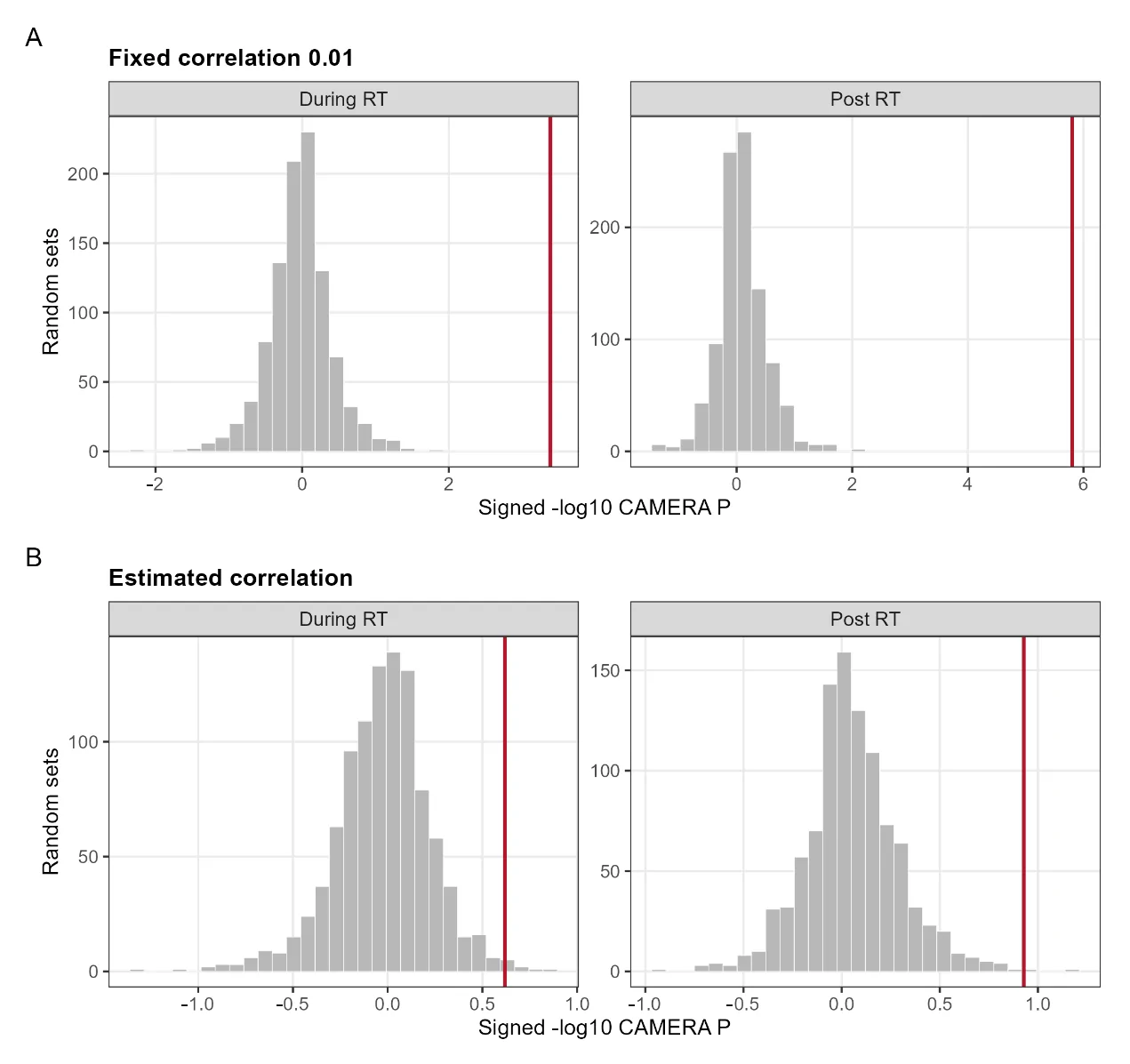
Expression-matched random gene-set analyses in the radiotherapy cohort. (**A**) Random-set comparisons using CAMERA with fixed intergene correlation of 0.01. (**B**) Corresponding comparisons with intergene correlation estimated separately for each gene set. Gray histograms show the signed −log10 CAMERA *p* values for 1000 random sets matched to the 63-gene periodontitis-upregulated set by gene-set size and mean-expression decile; red vertical lines indicate the observed periodontal set. Facets show comparisons during and after radiotherapy with baseline. Empirical two-sided *p* values were 0.000999 at both time points under fixed correlation and 0.03097 and 0.003996 during and after radiotherapy, respectively, under estimated correlation. These empirical *p* values use the random-set reference distribution and are distinct from the CAMERA *p* values. Matching did not preserve intergene correlation structure, cellular composition, or inflammatory-program membership; the analysis therefore does not establish periodontitis-specificity relative to other inflammatory conditions.

To assess whether the observed enrichment exceeded that of expression-matched gene sets, the 63-gene upregulated subset was compared with 1000 random sets matched for size and mean-expression decile. With intergene correlation fixed at 0.01, the observed absolute CAMERA statistic exceeded those of all random sets at both time points (two-sided empirical *p* = 0.000999; Figure 5A). When correlation was estimated separately for each set, the empirical *p* values were 0.0310 during radiotherapy and 0.00400 after treatment, with corresponding two-test FDR values of 0.0310 and 0.00799 (Figure 5B). The random sets had a median estimated correlation of approximately 0.043, compared with 0.199 for the observed set. Consequently, the empirical comparisons and the CAMERA significance tests addressed different reference distributions. The random-set results did not resolve the sensitivity to correlation assumptions or establish disease specificity.

### 2.6. Patient-Influence and Clinical Sensitivity Analyses

Leave-one-patient-out analyses retained positive estimates for the upregulated-component score at both radiotherapy time points, although the confidence intervals varied with patient inclusion (Figure S6A). Under the fixed-correlation CAMERA specification, the upregulated-set FDR ranged from 1.98 × 10^−4^ to 0.0963 during radiotherapy and from 2.62 × 10^−7^ to 0.00188 after treatment (Figure S6B). Thus, during-treatment enrichment did not remain significant in every omission analysis. These results describe sensitivity under the fixed-correlation specification and do not establish robustness to alternative correlation assumptions.

Clinical metadata reconciliation identified WHO mucositis grades for all eight patients. These values represented the maximum grade recorded during treatment rather than severity at each biopsy. Changes in the signed discovery score were not significantly associated with maximum WHO grade during radiotherapy (Spearman ρ = −0.408, exact *p* = 0.312; *n* = 8) or after radiotherapy (ρ = 0.617, exact *p* = 0.157; *n* = 7). The corresponding associations for the upregulated component were ρ = −0.482 (*p* = 0.226) and ρ = 0.767 (*p* = 0.0571). None of these four comparisons met FDR < 0.05 (Figure S7). Exploratory associations of discovery and replicated-subset score changes with dose at the second biopsy site or time from treatment initiation to that biopsy were also nonsignificant after correction.

A further sensitivity analysis retained the cisplatin classification used in the manuscript and deposited GEO metadata. Exclusion of patients labeled as receiving cisplatin left six baseline–during-treatment pairs and five baseline–post-treatment pairs. The signed discovery score changes were 0.472 during radiotherapy (95% CI −0.553 to 1.497, *p* = 0.290) and 0.537 after treatment (95% CI −0.383 to 1.457, *p* = 0.180; Figure S8). None of the eight score-by-time comparisons was significant. Fixed-correlation CAMERA enrichment of the upregulated subset remained detectable in this subset, with FDR values of 0.00108 and 4.88 × 10^−6^ during and after radiotherapy, respectively.

However, the original cohort publication assigned cisplatin exposure oppositely to the deposited GEO labels. Both classifications were retained in the metadata audit. The exclusion analysis therefore represents sensitivity to the manuscript-defined grouping and cannot be interpreted as a confirmed analysis of patients receiving radiotherapy without cisplatin.

### 2.7. Shared GO Biological-Process Enrichment Across Periodontal and Radiotherapy Cohorts

**Figure 6:**
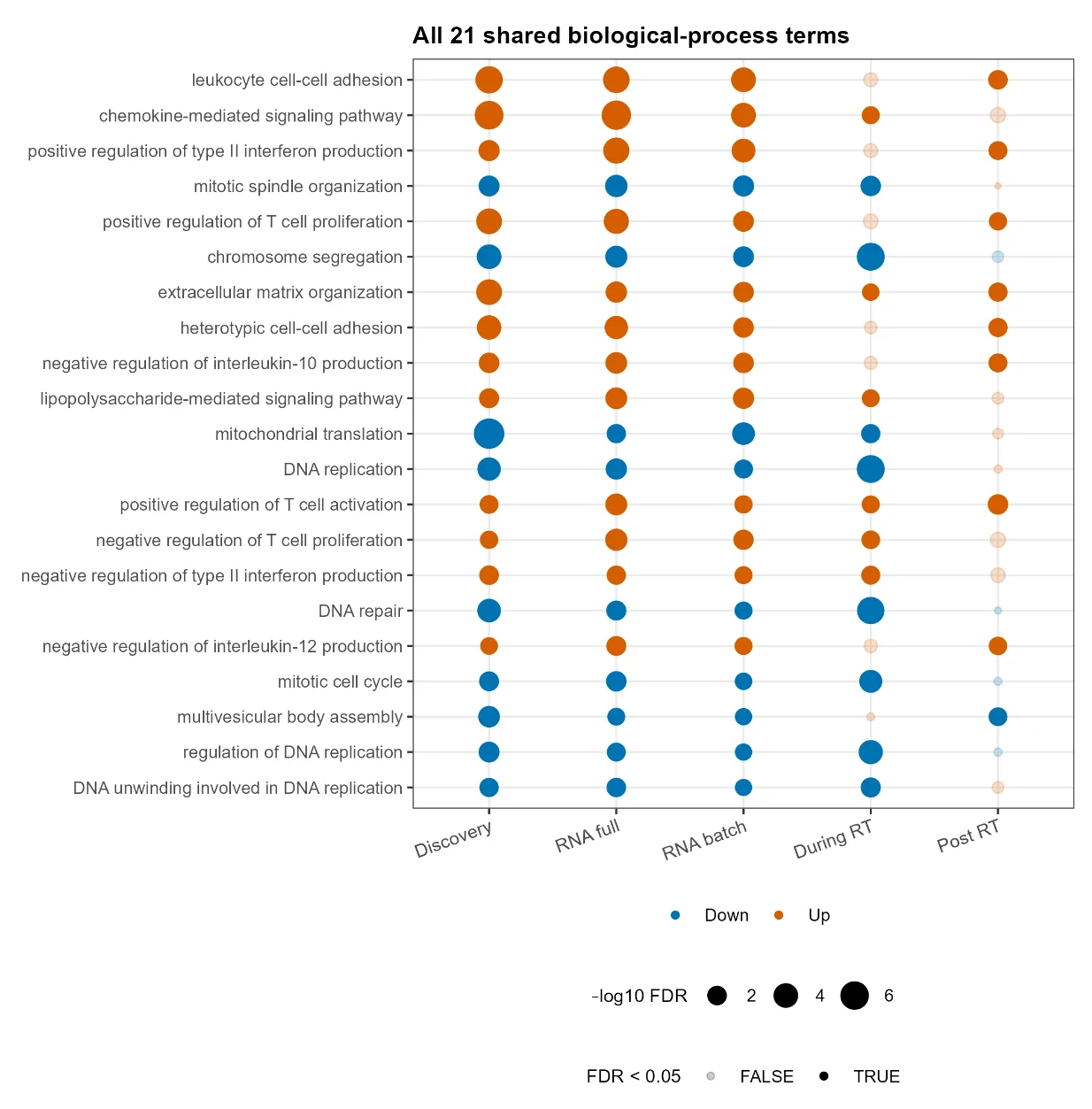
Cross-cohort enrichment of shared Gene Ontology biological processes. Dot plot of all 21 Gene Ontology (GO) biological-process terms meeting the shared-enrichment criteria. Columns represent the GSE10334 discovery model, the fully adjusted and batch-only GSE173078 models, and the during-radiotherapy and post-radiotherapy comparisons in GSE103412. Shared terms showed concordant enrichment directions and FDR < 0.05 in the discovery model, both RNA-sequencing models, and at least one radiotherapy comparison. Orange and blue denote positive and negative enrichment, respectively; circle size represents −log10 FDR, and faded circles indicate FDR ≥ 0.05 in the corresponding individual analysis. Enrichment was assessed using cameraPR with direct GO annotations and the original fixed-correlation specification of 0.01. The two RNA-sequencing models represent alternative analyses of the same cohort. Overlapping gene membership is documented in the Supplementary Materials, and individual GO terms should not be interpreted as independent mechanistic confirmations or direct measurements of functional activation or inhibition.

Rank-based GO biological-process analyses identified 21 terms with concordant enrichment directions and FDR < 0.05 in the discovery cohort, both evaluated RNA-sequencing models, and at least one radiotherapy time point (Figure 6). The two RNA-sequencing specifications represented alternative analyses of the same independent cohort. Twelve terms were shared exclusively with the during-radiotherapy comparison, comprising eight downward- and four upward-enriched terms; seven were shared exclusively with the post-radiotherapy comparison, comprising one downward- and six upward-enriched terms. Two upward-enriched terms were shared at both radiotherapy time points.

Upward-enriched terms included chemokine-mediated signaling during radiotherapy (radiotherapy FDR = 0.0281) and leukocyte cell–cell adhesion after treatment (FDR = 0.00985). Extracellular-matrix organization met the shared criteria at both time points, with radiotherapy FDR values of 0.0413 during treatment and 0.013 4 after treatment. Positive regulation of T-cell activation was also shared at both time points, with corresponding FDR values of 0.0281 and 0.00494.

Downward-enriched terms shared with the during-radiotherapy response included chromosome segregation, DNA replication, mitotic spindle organization, DNA repair, mitotic cell cycle, and mitochondrial translation. Their during-radiotherapy FDR values ranged from 2.25 × 10^−6^ to 0.0127. These findings described concordant transcriptional enrichment involving immune regulation, adhesion, extracellular-matrix organization, and cell-cycle-related processes.

Reanalysis reproduced all 8452 previously generated GO result entries to numerical precision. All 21 shared terms were retained in Figure 6, and their gene memberships and pairwise overlaps were exported to make annotation redundancy explicit. Because the terms share genes and were evaluated using the original rank-based enrichment specification, they should not be counted as independent mechanistic findings. Their enrichment directions describe transcriptomic correspondence rather than direct measurements of pathway activation, inhibition, or functional impairment.

### 2.8. Cellular Expression Proxies Account for Part of the Periodontal Score Association

**Figure 7:**
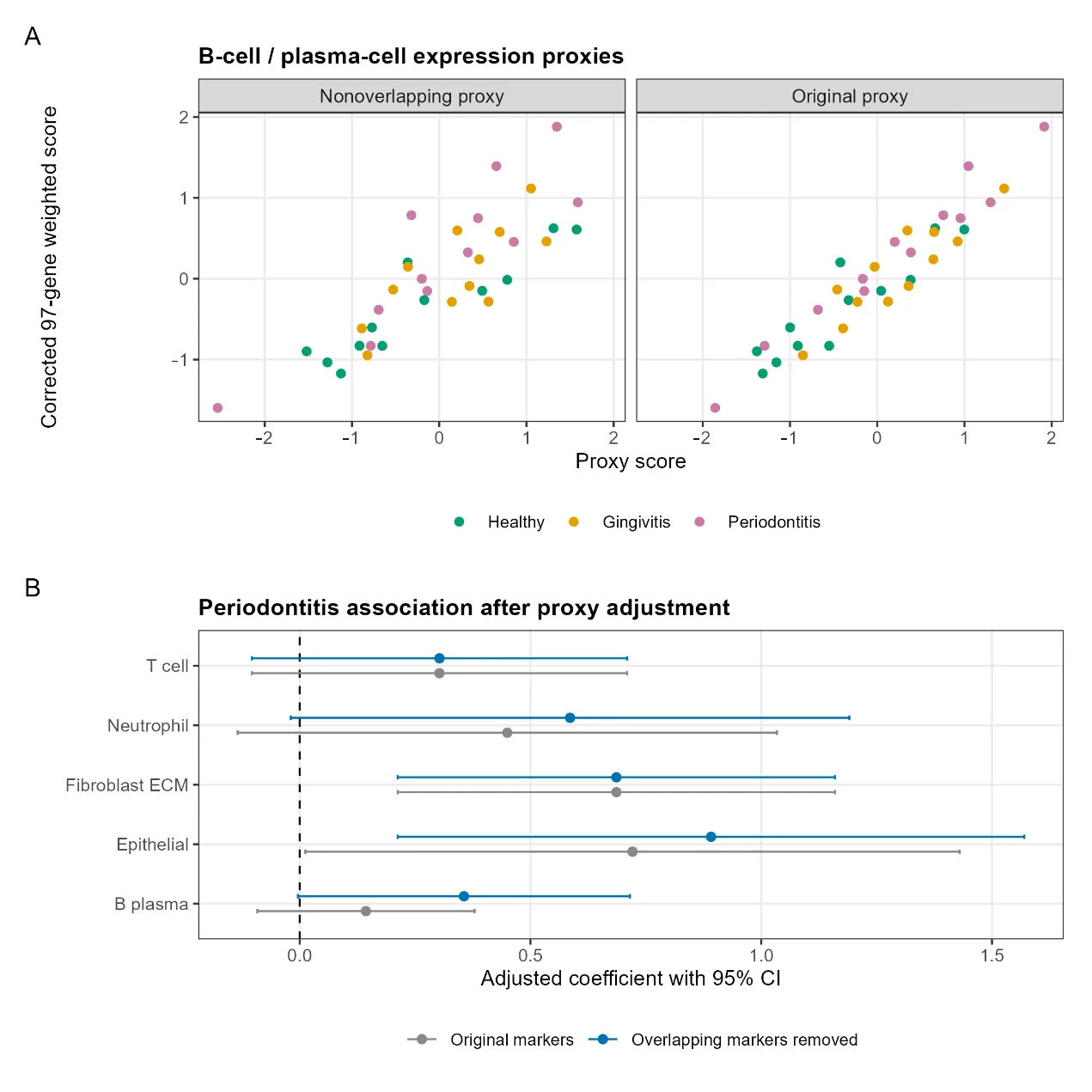
Sensitivity of the periodontal signature association to expression-derived cellular proxies. (**A**) Associations between the corrected equal-gene-weight 97-gene periodontal score and the B-cell/plasma-cell expression proxy, using nonoverlapping markers (left) or the original markers (right). Points represent individual RNA-sequencing samples (*n* = 36), and colors indicate clinical phenotype. Spearman correlations were 0.834 after removal of five shared genes and 0.938 with the original proxy. (**B**) Periodontitis-versus-health coefficients from models adjusted for batch, age, sex, and one cellular proxy at a time. Points indicate adjusted coefficients, and horizontal lines indicate 95% CIs; gray and blue indicate the original and nonoverlapping marker definitions, respectively. The dashed line marks a coefficient of zero. Proxies summarize marker-gene expression and are not independently measured cell fractions; attenuation after adjustment does not establish causal mediation by a cell population.

To assess the contribution of cellular expression patterns to the periodontal signature, the corrected equal-gene-weight 97-gene score was compared with expression-based cell-type proxies in GSE173078. The score was strongly correlated with the original B-cell/plasma-cell proxy (Spearman ρ = 0.938) and T-cell proxy (ρ = 0.797). Removal of the five genes shared by the B-cell/plasma-cell proxy and discovery signature reduced the former correlation to 0.834 (Figure 7A).

The association with the nonoverlapping B-cell/plasma-cell proxy persisted after rank-based adjustment for phenotype, batch, age, and sex (partial ρ = 0.846, approximate *p* = 2.05 × 10^−9^). Positive correlations were also observed separately within the healthy, gingivitis, and periodontitis groups (ρ = 0.902, 0.720, and 0.832, respectively). These findings indicated that the pooled association was not explained entirely by shared genes or differences between phenotype groups.

Adjustment for the original B-cell/plasma-cell proxy attenuated the periodontitis coefficient from 0.831 to 0.144 (95% CI −0.092 to 0.379, *p* = 0.222). Adjustment for the nonoverlapping proxy yielded a coefficient of 0.356 (95% CI −0.004 to 0.716, *p* = 0.0523; Figure 7B). In the nonoverlapping-proxy analyses, the periodontitis association remained significant after adjustment for the epithelial proxy (β = 0.891, 95% CI 0.212–1.570, FDR = 0.0296) or fibroblast/extracellular-matrix proxy (β = 0.686, 95% CI 0.212–1.160, FDR = 0.0296), but not after adjustment for the B-cell/plasma-cell, neutrophil, or T-cell proxies.

The attenuation associated with immune-cell proxies was consistent with a contribution of cellular composition or cellular state to the periodontal score. Because these proxies were derived from bulk expression data, the analyses did not quantify cell fractions or establish a causal cellular explanation for the signature.

## 3. Discussion

By integrating periodontal discovery, independent RNA-sequencing evaluation, and longitudinal radiotherapy analyses, this study examined transcriptional correspondence at the gene, signature-score, and biological-process levels. The periodontal signature showed concordant effect directions in the independent cohort, and its score remained associated with periodontitis across the two evaluated score models. However, gene-level replication was sensitive to covariate adjustment and sample inclusion. In the radiotherapy cohort, neither the mapped periodontal genes nor longitudinal signature scores met their respective significance criteria. Enrichment of the periodontitis-upregulated set was detected under the fixed-correlation CAMERA specification but did not remain significant when intergene correlation was estimated from the data. Together, these findings indicate partial transcriptional correspondence whose interpretation depends on the analytical level and statistical assumptions, without establishing that periodontitis modifies radiation injury or predicts clinical mucositis.

The periodontal findings provide a foundation for this comparison, although their reproducibility requires qualification. Strong directional agreement across cohorts and platforms, together with an independently supported score association, indicates that the discovery signal was not confined to the original sampling design. The corrected 97-gene alternative score also showed a stronger association with periodontitis than expression-matched random signatures. Nevertheless, 23 discovery genes met the replication criteria in the fully adjusted model and none did so in the batch-only model. All 23 retained positive effect estimates in the leave-one-sample-out analyses, but no gene met the replication thresholds in every iteration. Effect and dispersion diagnostics, together with a separate voom-based assessment of standard errors, did not support a uniform improvement in statistical precision as the explanation for the larger number of significant genes after adjustment. These findings distinguish consistent effect direction from stable threshold-based replication and do not establish one covariate specification as definitively correct.

Cellular composition or cellular transcriptional state may contribute to the periodontal signal. The score remained strongly associated with the B-cell/plasma-cell proxy after removal of the five shared genes, adjustment for phenotype and other covariates, and examination within phenotype groups. Thus, shared markers and between-group differences did not fully account for the observed correlation. Adjustment for the nonoverlapping proxy nevertheless attenuated the periodontal coefficient, with its confidence interval including zero. This finding is compatible with a contribution from immune-cell abundance or state, but residual correlation between expression-derived measurements and potential adjustment for disease-associated biology precludes a causal interpretation. Previous bulk-tissue decomposition and single-cell analyses document distinct epithelial, fibroblast, B-cell, and plasma-cell contributions to gingival expression profiles [11,12]. Independent gingival RNA-sequencing and treatment-response studies likewise show that the measured tissue transcriptome reflects several changing compartments [13,14]. The signature is thus best interpreted as a tissue-level expression pattern whose cellular determinants remain unresolved.

The radiotherapy findings require a distinction between the questions addressed by the different analyses. The absence of significant effects among 18 mapped replicated periodontal genes and enrichment of the broader 63-gene upregulated set cannot be attributed solely to greater power from aggregating weak signals: these analyses also differ in gene membership and statistical hypothesis. CAMERA evaluates a set relative to genes outside it, whereas longitudinal score models assess changes in predefined expression summaries across patients [15]. Separating the upregulated and downregulated score components did not yield significant longitudinal changes after correction, indicating that the nonsignificance of the signed score cannot be explained solely by its combination of directional components. The wide confidence intervals indicate limited precision rather than evidence that a biological response is absent.

The correlation sensitivity analysis materially qualifies the original enrichment finding. The estimated residual correlation of the upregulated set was approximately 0.199, compared with the fixed value of 0.01 used originally. Allowing CAMERA to estimate this correlation changed the upregulated-set FDR values to 0.360 during radiotherapy and 0.177 after treatment. FRY directional tests also did not meet the significance threshold. Mixed-direction FRY results support changes within some gene sets but do not demonstrate a coherent shift toward the periodontal expression pattern. Consequently, the data do not support describing the complete periodontal signature as consistently activated across patients. The fixed-correlation findings remain informative as results under a specified analytical assumption, rather than as evidence robust to all evaluated alternatives.

The new random-set analyses provide a complementary assessment of the background against which correspondence was evaluated. In the radiotherapy cohort, the upregulated set showed a more extreme CAMERA statistic than expected from size- and expression-matched random sets under both correlation specifications. This empirical result does not contradict the nonsignificant CAMERA results obtained with estimated correlation, because the two procedures use different reference distributions. Moreover, the random sets had a lower median estimated correlation than the periodontal set, and matching did not preserve cellular composition or inflammatory-program membership. The analysis therefore does not establish specificity relative to other inflammatory conditions. Comparisons with independent inflammation- and injury-associated signatures would be needed to distinguish a periodontitis-specific relationship from a more general oral tissue response.

The shared biological processes provide a plausible context for this qualified overlap. Chemokine signaling, leukocyte adhesion, T-cell regulation, and extracellular-matrix organization connect immune recruitment with changes in the tissue microenvironment. Human oral mucosal single-cell profiling, spatial profiling of human gingiva, neutrophil atlases, and experimental studies of epithelial–fibroblast–neutrophil signaling support the importance of location and cell context [16,17,18,19]. In a separate human radiotherapy cohort, immunohistochemistry documented changes in oral-mucosal macrophage subsets, and an irradiated three-dimensional oral-mucosal model showed inflammatory transcriptional responses [20,21]. These observations provide a cellular framework within which the present bulk-tissue findings can be interpreted, although they do not establish that identical cell populations generate the periodontal and radiotherapy signals. Anatomical differences between gingiva and buccal mucosa and the dependence of bulk expression on both cellular composition and cellular state further constrain transfer of a signature across tissues [22,23].

All 21 concordantly enriched GO terms were retained in the revised analysis, with gene-membership overlap reported explicitly. Agreement across the two periodontal cohorts and at least one radiotherapy comparison is descriptive cross-cohort enrichment under the specified ranking, annotation, and testing procedures. The two RNA-sequencing models analyze the same cohort and do not constitute additional independent replications. Likewise, overlapping GO terms should not be counted as separate mechanistic confirmations, and the rank-based GO analysis retained its original fixed-correlation specification. The pathway findings therefore do not independently resolve the correlation sensitivity observed for the transferred periodontal signature.

The negatively enriched processes warrant similarly careful interpretation. Gene sets related to chromosome segregation, DNA replication, mitotic progression, DNA repair, and mitochondrial translation showed concordant negative enrichment in periodontitis and during radiotherapy. These findings were accompanied by lower expression of proliferation- and histone-associated transcripts in the radiotherapy cohort, but they do not directly measure proliferative capacity or DNA-repair function. Earlier human biopsy data documented radiation-associated changes in oral epithelial cellularity and proliferation; experimental work in mice has also implicated epithelial stem-cell DNA double-strand break repair in mucosal injury [24,25]. The original investigation of this cohort reported radiation-associated changes involving apoptosis, DNA damage, repair, and proliferation without a correlation with clinical mucositis severity; because it used the same underlying cohort, it provides context rather than independent confirmation [3]. Increased expression of selected DNA-damage-response-associated genes alongside negative enrichment of broader DNA-repair-related sets may reflect differences among transcriptional modules, sampling times, or cellular composition. These explanations remain hypotheses and should not be reduced to a claim of globally impaired repair activity.

The time-point-specific enrichment findings also suggest questions for further investigation. Chemokine-related processes met the shared-enrichment criteria during treatment, leukocyte adhesion did so after treatment, and T-cell activation and extracellular-matrix organization were represented at both time points. However, significance at one time point and not another does not establish a difference between those time points. Although direct longitudinal contrasts were included in the expression analyses, the overlap of GO significance categories alone cannot define a transition from acute inflammation to repair. More frequent sampling would be needed to characterize transient, sustained, and delayed responses. Likewise, the absence of significant competitive enrichment in the periodontitis-downregulated set does not demonstrate that its constituent genes are unaffected by irradiation.

Recovery of clinical metadata enabled an exploratory assessment of clinical relevance, but did not establish a relationship between the signatures and mucositis severity. None of the tested associations with maximum WHO grade remained significant after correction; the dose and biopsy-timing analyses were also nonsignificant. Maximum WHO grade was not measured concurrently with each biopsy, limiting correspondence between clinical and molecular observations. In addition, cisplatin exposure was classified inconsistently in the deposited metadata and the original publication. The exclusion analysis therefore tests sensitivity to the manuscript-defined grouping and cannot be interpreted as a verified radiotherapy-only analysis or as evidence that chemotherapy confounding has been removed. A prior clinical pilot explored periodontitis and radiation-associated mucositis without establishing a definitive association [26]. Prospective radiotherapy cohorts have documented periodontal, dental, and other oral sequelae [27,28,29], but these outcomes do not establish the molecular relationship examined here.

Several limitations define the scope of this study. First, periodontal and radiotherapy responses were studied in separate cohorts, and periodontal status was unavailable for the radiotherapy patients; the design therefore cannot test whether baseline periodontitis modifies radiation-associated mucosal injury. Second, the eight during-treatment and seven post-treatment pairs limit precision and the stability of sensitivity and clinical analyses. Third, anatomical and platform differences, incomplete cross-platform gene representation, bulk-tissue measurements, and expression-derived cellular proxies prevent a definitive separation of shared cellular abundance from shared regulation within cells. Fourth, covariate- and correlation-dependent results, overlapping pathway annotations, and multiple exploratory analyses restrict the strength of confirmatory inference; correction within separate testing families does not provide a study-wide error guarantee. Finally, sparse biopsy timing, the absence of biopsy-specific severity grades, and unresolved treatment annotations limit clinical interpretation. These data neither validate a predictive biomarker nor establish that treating periodontitis would reduce radiation-induced mucositis.

The most direct next step is a prospective study measuring periodontal status, verified treatment exposure, serial mucosal expression, and mucositis severity within the same patients. Such a design could determine whether the overlapping programs precede injury, accompany its severity, or persist during recovery, and whether these relationships vary with baseline periodontal disease. Cell-resolved profiling and protein-level measurements would help distinguish altered cellular composition from changes within cells and test the functional relevance of the transcriptional findings. Comparison with other inflammatory and injury-associated signatures would further clarify specificity. The present study identifies candidate areas of transcriptional correspondence while defining the statistical and clinical conditions that must be resolved before mechanistic or translational conclusions can be drawn.

## 4. Materials and Methods

### 4.1. Study Design and Transcriptomic Datasets

Three publicly available human transcriptomic datasets were integrated to examine the correspondence between periodontitis-associated gene expression and oral mucosal responses to radiotherapy. The analytical workflow comprised discovery of a periodontal transcriptional signature, evaluation in an independent RNA-sequencing cohort, and assessment of its gene-level, signature-level, and biological-process-level correspondence with longitudinal radiotherapy responses. Each dataset was processed and modeled independently; expression matrices from different platforms were not pooled.

GSE10334 served as the periodontal discovery cohort and comprised 247 gingival tissue profiles from 90 individuals, including 183 periodontitis-affected sites and 64 clinically healthy sites. Participants in the original study were recruited at the Columbia University College of Dental Medicine, New York, NY, USA, between November 2004 and April 2007. This interval represents the reported recruitment period rather than individual specimen collection dates. Analyses accounted for multiple samples obtained from the same individual. Clinically healthy sites in this cohort were distinguished from gingival samples obtained from healthy participants in the independent cohort.

GSE173078 provided an independent RNA-sequencing cohort of 36 gingival samples, with 12 samples each from healthy participants, patients with gingivitis, and patients with periodontitis. The original study was approved by the Institutional Review Board of Columbia University Irving Medical Center, New York, NY, USA (protocol AAAP6006). Gingival specimens were obtained during clinically indicated oral surgical procedures. The calendar period of participant recruitment and specimen collection was not specified in the publicly available source information reviewed. Clinical phenotype, sequencing batch, age, and sex were obtained from the accompanying metadata.

GSE103412 was used to evaluate longitudinal oral mucosal responses to radiotherapy. Patients in the original study were recruited at the Department of Maxillofacial Surgery, Aalborg University Hospital, Aalborg, Denmark, between 1 September 2010, and 30 April 2013. Healthy-control specimens originated from a previous study; therefore, this recruitment interval should not be interpreted as the collection period for all specimens in the dataset. Of its 64 expression profiles, 32 were derived from buccal mucosa: nine healthy-control profiles and 23 patient profiles, comprising eight baseline, eight during-radiotherapy, and seven post-radiotherapy samples. Longitudinal analyses were restricted to the 23 patient profiles, providing eight baseline–during-treatment pairs and seven baseline–post-treatment pairs. Healthy controls were included in descriptive quality assessments but were not treated as patient baseline observations.

Expression data, platform annotations, and sample metadata were obtained from the Gene Expression Omnibus. Sample identifiers were reconciled between expression matrices and metadata before analysis. This study involved secondary analysis of publicly available data; no participants were recruited and no additional specimens were collected. Analyses added during revision were treated as sensitivity or exploratory analyses and were not described as prospectively registered.

### 4.2. Expression Preprocessing, Identifier Harmonization and Quality Assessment

For GSE10334 and GSE103412, the deposited processed microarray expression matrices were used. Expression distributions and numerical ranges were inspected, and missing or non-finite values were assessed. Probe or transcript identifiers were mapped to gene symbols using the corresponding platform annotations. Entries without an eligible gene assignment or with ambiguous mappings were excluded. When multiple retained probes mapped to the same gene, their processed expression values were averaged to obtain gene-level matrices.

For GSE173078, deposited gene-level read counts were analyzed using edgeR. Low-expression genes were filtered using filterByExpr with a design containing sequencing batch and phenotype, retaining 15,849 genes. Library composition was normalized using the trimmed mean of M values method. Differential-expression models used count data, whereas normalized log_2_ counts per million, calculated with a prior count of 2, were used for visualization and signature scoring.

Cross-cohort matching used gene symbols after trimming whitespace and harmonizing letter case. Original identifiers and harmonized matching keys were retained in an audit table, and expression matrices were checked for duplicate matching keys. This procedure resolved the C16ORF54/C16orf54 discrepancy without introducing unrestricted gene-alias substitutions. Genes absent from a cohort’s eligible expression matrix were recorded as unmapped and omitted from the relevant signature.

Quality assessment included sample-wise expression distributions and principal-component analysis based on the 2000 most variable genes or transcript clusters. The revised quality-control figures were generated from the processed gene-level matrices. Sample patterns were examined in relation to phenotype, batch, and sampling time. All discovery-cohort samples were retained in the primary analysis; exclusion of the three samples flagged in the original principal-component assessment was evaluated separately.

### 4.3. Identification of the Periodontal Discovery Signature

Differential expression between periodontitis-affected and clinically healthy gingival sites in GSE10334 was assessed using limma [30]. Clinical site status was included in the design matrix. Within-patient correlation was estimated using duplicateCorrelation, and the consensus correlation was incorporated into a patient-blocked linear model. Gene-wise statistics were moderated using the empirical Bayes procedure.

Two sensitivity analyses assessed sample influence and the handling of repeated measurements. First, the patient-blocked model was refitted after excluding the three samples flagged by the original principal-component assessment. Second, for the 64 individuals contributing both affected and clinically healthy sites, expression was averaged within each patient and site category. Patient-specific affected-minus-healthy differences were then analyzed to estimate the mean within-patient effect.

Differentially expressed genes were defined by the joint criteria of |log_2_ fold change (FC)| > 1 and Benjamini–Hochberg false discovery rate (FDR) < 0.05. The primary model defined the discovery signature, with upregulated and downregulated genes retained as separate components. Agreement across analyses was assessed using selected-gene overlap, effect-direction concordance, and Pearson correlations between genome-wide log_2_FC estimates.

### 4.4. Independent Evaluation and Model-Sensitivity Analyses in the Periodontal RNA-Sequencing Cohort

Differential expression in GSE173078 was analyzed using edgeR quasi-likelihood models with robust dispersion estimation and robust quasi-likelihood fitting. The primary model included phenotype, sequencing batch, age, and sex, with healthy gingiva as the reference phenotype. Age was standardized as a continuous covariate. Design-matrix rank and residual degrees of freedom were inspected to confirm model estimability.

The primary contrast compared periodontitis with healthy gingiva. Additional contrasts compared gingivitis with healthy gingiva and periodontitis with gingivitis. Three alternative models included phenotype with batch alone, batch and age, or batch and sex. Each model used the same filtered gene universe, and gene-level *p* values were adjusted across all tested genes within the corresponding comparison.

Discovery genes were matched to the RNA-sequencing results using harmonized symbols. Cross-cohort agreement was quantified using Spearman correlations between log_2_FC estimates and the proportion of mapped genes with concordant effect directions. Replication required concordant direction, |log_2_FC| > 1, and FDR < 0.05 in the fully adjusted RNA-sequencing model. The same criteria were evaluated under the alternative covariate specifications.

The discovery signature was defined in GSE10334 before its evaluation in GSE173078. The 23-gene replicated subset was selected using the fully adjusted RNA-sequencing results and was subsequently held fixed for influence analyses and evaluation in the radiotherapy cohort.

To assess individual-sample influence, the fully adjusted model was refitted after omitting each of the 36 RNA-sequencing samples in turn. The original filtering universe, library sizes, and normalization factors were retained, while dispersion estimates and model coefficients were re-estimated. For each of the original 23 genes, effect direction, log_2_FC, FDR, and the frequency of meeting the replication criteria were recorded. These analyses assessed influence conditional on the original preprocessing rather than repeating the complete signature-discovery procedure.

Model diagnostics included design leverage, negative-binomial dispersion, and quasi-likelihood posterior dispersion. Because edgeR quasi-likelihood F tests do not directly supply conventional Wald standard errors, a separate voom–limma analysis was fitted for effect and precision comparisons. Voom precision weights were calculated for each design, followed by linear modeling and robust empirical Bayes moderation. Model-specific moderated standard errors and 95% confidence intervals were reported separately and were not combined with edgeR coefficients as though they arose from the same model.

### 4.5. Longitudinal Differential-Expression Analysis in the Radiotherapy Cohort

Gene expression in GSE103412 was modeled using categorical sampling time and fixed patient effects. Sampling time comprised baseline, during radiotherapy, and after radiotherapy. Patient effects accounted for stable between-patient differences across repeated observations.

The original during-radiotherapy-versus-baseline and post-radiotherapy-versus-baseline differential-expression tables were obtained using limma linear models followed by standard empirical Bayes moderation. These tables defined the radiotherapy-responsive gene sets displayed in the heatmap and volcano plots. A subsequent analysis used robust empirical Bayes moderation with an intensity-dependent variance trend to estimate effects and confidence intervals for mapped periodontal genes and to evaluate the unified three-time-point contrasts, including post-radiotherapy versus during-radiotherapy expression. The two moderation specifications were distinguished in the analysis records.

Genes meeting both FDR < 0.05 and |log_2_FC| > 1 were classified as differentially expressed. Counts based on FDR < 0.05 alone were summarized separately. All available patient profiles were retained in the joint longitudinal model; missing follow-up observations were not imputed.

Sensitivity analyses used complete baseline–follow-up pairs. An additional exclusion analysis followed the cisplatin labels used in the original manuscript and deposited GEO metadata, retaining six baseline–during-treatment pairs and five baseline–post-treatment pairs. Gene-level models in the revised exclusion analysis used robust, trend-dependent empirical Bayes moderation. Conflicting cisplatin classifications in the original cohort publication were preserved separately, as described in Section 4.12.

### 4.6. Evaluation of Periodontal Gene Sets in Irradiated Oral Mucosa

Three discovery-derived sets were evaluated in GSE103412: periodontitis-upregulated genes, periodontitis-downregulated genes, and their union. After mapping, these sets contained 63, 28, and 91 genes, respectively. The reference universe consisted of all genes in the processed patient oral-mucosa expression matrix.

Gene-set associations with the during-radiotherapy and post-radiotherapy contrasts were tested using CAMERA with the patient-and-time design. The original specification fixed intergene correlation at 0.01. A sensitivity analysis estimated intergene correlation from the data by setting inter.gene.cor = NA. Both specifications were reported to assess the dependence of enrichment inference on this assumption.

FRY was used as a complementary self-contained test with the same expression data, gene sets, design, and contrasts. Directional and mixed-direction results were retained separately. Mixed-direction significance was interpreted as evidence of expression changes within a set, without requiring a concordant shift in the direction observed in periodontitis.

Benjamini–Hochberg correction was applied across the three gene sets within each time-point contrast and testing procedure. CAMERA results under the two correlation specifications were treated separately; directional and mixed-direction FRY results also formed separate testing families.

The 18 mapped genes from the replicated periodontal subset were evaluated individually, with log_2_FC estimates, 95% confidence intervals, and gene-level FDR values reported separately from set-level results. Gene-level FDR values were derived from the corresponding full expression analysis rather than recalculated only across these 18 genes.

Patient influence was assessed by removing all observations from one patient at a time and refitting the gene-set analyses. The original gene-set definitions were retained. The reported leave-one-patient-out CAMERA summaries used the fixed-correlation specification and were distinguished from the data-estimated-correlation sensitivity analysis.

### 4.7. Calculation and Modeling of Sample-Level Periodontal Signature Scores

The primary periodontal score was calculated from gene-wise standardized expression values. For each gene, expression was centered and scaled across samples in the corresponding scoring dataset:

$$z_{gs}=\frac{x_{gs}-{\bar{x}}_{g}}{s_{g}}$$

where $x_{gs}$ denotes the expression of gene g in sample s, and ${\bar{x}}_{g}$ and $s_{g}$ denote the gene-specific mean and standard deviation, respectively.

The signed discovery score was defined as

$$S_{s}=\frac{1}{\left|U\right|}\sum_{g\in U}z_{gs}-\frac{1}{\left|D\right|}\sum_{g\in D}z_{gs}$$

where U and D denote the mapped upregulated and downregulated discovery genes, respectively. This formulation assigns equal aggregate weight to the two directional components. Because the replicated subset contained only upregulated genes, its score was calculated as the mean standardized expression of the mapped genes.

The primary discovery score included 97 mapped genes in GSE173078 and 91 in GSE103412. The replicated-subset score included 18 of the 23 genes in GSE103412. Genes without expression measurements were omitted rather than assigned zero expression. Because standardization was performed separately within each dataset, the scores were interpreted as relative expression summaries and were not compared directly across cohorts.

In GSE173078, associations between signature scores and phenotype were evaluated using linear models adjusted for batch, age, and sex; a batch-adjusted model served as a sensitivity analysis. In GSE103412, scores were analyzed using linear models with categorical sampling time and fixed patient effects. Contrasts estimated changes during radiotherapy versus baseline, after radiotherapy versus baseline, and after versus during radiotherapy. Model estimates were reported with 95% confidence intervals and two-sided *p* values.

Estimates were reported with model-based 95% confidence intervals and two-sided *p* values. For the revised component analysis, Benjamini–Hochberg correction was applied across the eight comparisons formed by four scores—upregulated, downregulated, signed discovery, and replicated subset—and two follow-up-versus-baseline contrasts.

Leave-one-patient-out score analyses retained the original standardized scores and removed all observations from the omitted patient before refitting the model. This preserved the score definition while evaluating sensitivity to patient inclusion.

### 4.8. Paired Sensitivity Analyses of Signature Changes

Within-patient changes were calculated as follow-up minus baseline scores. Complete pairs were summarized using the mean, median, standard deviation, and numbers of patients with positive or negative changes. These summaries were distinguished from estimates obtained from the joint three-time-point model.

Exact two-sided sign-flip tests compared the observed absolute mean change with the distribution obtained by enumerating all 2^n^ sign assignments to the n paired differences. This procedure evaluates a symmetric null distribution of paired changes. In the original paired sensitivity analyses, Wilcoxon signed-rank tests were also calculated using a normal approximation with continuity correction.

The original paired analyses evaluated the signed discovery and replicated-subset scores at two follow-up time points in both the full sample and manuscript-defined cisplatin-exclusion subset. Benjamini–Hochberg correction was applied across these eight comparisons separately for the sign-flip and Wilcoxon procedures.

The revised cisplatin-exclusion analysis additionally evaluated all four score formulations. For each follow-up comparison, a patient-fixed-effect model was fitted to complete baseline–follow-up pairs, and an exact sign-flip *p* value was calculated from the corresponding differences. Correction was applied across the eight score-by-time comparisons separately for model-based and sign-flip *p* values. Standardization remained fixed to the full 23-profile patient scoring dataset. Nonsignificance was not interpreted as evidence of equivalence or absence of an effect.

### 4.9. Expression-Matched Random Gene-Set Analysis

A complementary equal-gene-weight score was defined as

$$S_{s}^{\mathrm{alt}}=\frac{1}{N}\sum_{g=1}^{N}\left(d_{g}z_{gs}\right)$$

where $z_{gs}$ denotes the standardized expression of gene g in sample s, as defined in Section 4.7; N is the number of mapped signature genes; and $d_{g}$ equals +1 for discovery-upregulated genes and −1 for discovery-downregulated genes. Unlike the primary signed score, this formulation assigns equal absolute weight to each gene rather than equal aggregate weight to each directional component. After identifier harmonization, it included 97 genes in GSE173078: 67 upregulated and 30 downregulated. The earlier case-sensitive 96-gene implementation was retained only for a mapping-impact comparison.

For the RNA-sequencing analysis, genes were assigned to deciles using ranks of mean normalized log_2_ counts per million. Signature genes were excluded from the sampling background. Each of 1000 random sets was sampled without replacement within expression strata to reproduce the observed signature’s size and decile composition. Direction labels were randomly assigned while preserving the numbers of positive and negative labels. Random sets could overlap with one another, but no set contained duplicate genes.

Observed and random scores were analyzed using the same linear model incorporating phenotype, sequencing batch, age, and sex. The two-sided empirical *p* value was calculated as

$$P_{emp}=\frac{1+\sum_{b=1}^{B}I(|{\hat{\beta }}_{b}|\geq |{\hat{\beta }}_{\mathrm{obs}}|)}{B+1}$$

where B = 1000, ${\hat{\beta }}_{\mathrm{obs}}$ is the estimated periodontitis-versus-health coefficient for the observed signature, and ${\hat{\beta }}_{b}$ is the corresponding coefficient for random set b. The indicator function I(·) equals 1 when the condition is satisfied and 0 otherwise.

A separate random-set analysis was conducted in GSE103412 for the 63-gene periodontitis-upregulated subset. Background genes were stratified by mean expression across the 23 patient profiles, and all mapped discovery-signature genes were excluded from sampling. The 1000 random sets matched the observed subset in size and expression-decile composition.

Each observed or random set was evaluated using CAMERA with the same patient-and-time model. The comparison statistic was the signed negative log_10_ CAMERA *p* value, with positive values indicating upward enrichment. Two-sided empirical *p* values compared the absolute observed and random statistics using the same add-one correction described above, with the CAMERA-derived statistic replacing the regression coefficient. Analyses were conducted with intergene correlation fixed at 0.01 and repeated on the same sets with correlation estimated separately for each set. Empirical *p* values were adjusted using the Benjamini–Hochberg procedure across the two time points within each correlation specification.

The revised random-set analyses used seed 20261022. Participant labels were not permuted. Matching did not preserve intergene correlation, cellular composition, or membership in disease-specific inflammatory programs; empirical enrichment relative to these backgrounds was therefore distinguished from evidence of periodontitis specificity.

### 4.10. Expression-Based Cellular Proxy Analyses

Exploratory cellular proxy analyses in GSE173078 assessed the relationship between the corrected equal-gene-weight score and expression patterns associated with major cellular compartments. Candidate markers for B cells/plasma cells, neutrophils, T cells, epithelial cells, and fibroblasts/extracellular matrix are listed in Table 2.

Each proxy was calculated as the mean gene-wise standardized expression of its available markers. A proxy was evaluated only when at least three markers were represented. Two versions were analyzed: the original marker set and a nonoverlapping set obtained by removing all genes shared with the discovery signature. The original B-cell/plasma-cell proxy shared CD19, CD27, CD79A, MZB1, and TNFRSF17 with the signature. Actual mapped marker membership was recorded for both versions.

Spearman correlations were calculated across all 36 samples and separately within each phenotype group. To assess whether pooled associations reflected between-group differences, score and proxy ranks were independently residualized against phenotype, batch, standardized age, and sex. Their residual Pearson correlation was reported as the adjusted rank correlation. Approximate two-sided *p* values used a t distribution with n − rank(X) − 1 degrees of freedom, where X was the covariate design matrix. Benjamini–Hochberg correction was applied across the five adjusted correlations separately for original and nonoverlapping proxies. Within-group correlations were treated as descriptive.

Each proxy was also added individually to the phenotype-, batch-, age-, and sex-adjusted score model. The periodontitis coefficient, 95% confidence interval, and *p* value were extracted. Correction was applied across the five proxy-adjusted models separately for each marker-set version.

These analyses assessed sensitivity to expression-derived cellular patterns. The proxies were not interpreted as measured cell fractions, and coefficient attenuation was not treated as proof of cellular mediation.

### 4.11. Cross-Cohort Analysis of GO Biological Processes

GO biological-process enrichment used complete gene-level results rather than threshold-selected differentially expressed genes. Analyses included the GSE10334 periodontal comparison, fully adjusted and batch-only GSE173078 comparisons, and during-radiotherapy and post-radiotherapy comparisons in GSE103412.

Directional statistics were obtained from moderated t statistics for limma analyses and from sign(log_2_FC)√F for edgeR quasi-likelihood analyses. Gene symbols were harmonized, and entries without finite statistics were excluded. Where duplicate symbols occurred, the entry with the largest absolute statistic was retained. Each comparison’s complete eligible statistic vector defined its reference universe.

Gene sets were constructed from direct human gene-to-GO biological-process annotations in org.Hs.eg.db using AnnotationDbi. Direct GO associations were used rather than ancestor-propagated GOALL membership. Sets were intersected with each comparison’s gene universe, and those containing 15–300 mapped genes were tested using limma cameraPR with use.ranks = TRUE and its default intergene correlation of 0.01. FDR correction was applied across all eligible terms separately within each model.

A term was classified as shared when it showed FDR < 0.05 and concordant enrichment direction in GSE10334, both GSE173078 model specifications, and at least one radiotherapy comparison. Shared terms were classified as meeting these criteria during radiotherapy only, after radiotherapy only, or at both time points. These categories described the statistical overlap criteria rather than biological exclusivity. The two RNA-sequencing models were treated as alternative specifications of one cohort.

All 21 shared terms were retained in the revised figure. The original descriptive redundancy analysis represented each term by itself and its biological-process ancestors from GO.db, calculated pairwise Jaccard similarity, and used a greedy grouping threshold of 0.70. This grouping did not determine which terms were displayed in the revised figure.

An additional audit quantified overlap between the actual annotated gene memberships of shared terms. Pairwise intersection sizes and Jaccard indices were exported, together with term membership after restriction to each model’s gene universe. Complete ranked statistics, annotation memberships, and enrichment results were retained for reproducibility. Shared terms were not assumed to constitute independent findings, and enrichment direction was not interpreted as a direct measurement of pathway activity or tissue function.

### 4.12. Clinical Metadata Reconciliation and Exploratory Associations

Radiotherapy metadata were reconciled with the original cohort publication at the patient level. Patient identifiers were matched across sources, and maximum WHO mucositis grade, cisplatin classification, biopsy-site radiation dose, and reported sampling intervals were recorded with their provenance.

The WHO variable represented the maximum mucositis grade during treatment and was not assigned to individual biopsy visits as contemporaneous severity. For each patient, changes in the signed discovery and upregulated-component scores were calculated relative to baseline. Associations with maximum WHO grade were assessed using Spearman correlation separately during and after radiotherapy.

Exact two-sided permutation *p* values were calculated by enumerating all patient-level permutations of the clinical ranks, retaining tied ranks, and comparing absolute permuted correlations with the observed correlation. Benjamini–Hochberg correction was applied across the four comparisons formed by two scores and two follow-up times.

A separate exploratory family assessed during-treatment changes in the signed discovery and replicated-subset scores against radiation dose at the second biopsy site and time from treatment initiation to that biopsy. The same exact rank-permutation procedure was used, with correction across four comparisons. Reported intervals between the second and third biopsies were not interpreted as time since radiotherapy completion.

The cisplatin classifications in GEO and the original publication were contradictory. Both were retained without overwriting either source. To reproduce the manuscript’s grouping, the exclusion analysis used the deposited GEO labels, yielding six during-treatment and five post-treatment pairs. Under the publication’s classification, only two patients were labeled as not receiving cisplatin; their changes were retained descriptively. No causal comparison between radiotherapy and chemoradiotherapy was attempted.

All clinical analyses used patients as the independent units. No prediction model was fitted, and exploratory associations were not treated as validation of a clinical biomarker.

### 4.13. Statistical Reporting, Visualization, and Computational Reproducibility

All analyses were performed in R version 4.4.2 (R Foundation for Statistical Computing, Vienna, Austria; https://www.r-project.org, accessed on 22 August 2026) with Bioconductor release 3.20 (https://bioconductor.org, accessed on 22 August 2026). Differential expression used limma version 3.62.2 and edgeR version 4.4.2; gene-set tests used CAMERA, cameraPR, and FRY from limma version 3.62.2; and low-expression filtering used filterByExpr from edgeR version 4.4.2. Gene annotations were obtained from org.Hs.eg.db version 3.20.0 through AnnotationDbi version 1.68.0, with GO.db version 3.20.0. Public datasets were retrieved from the NCBI Gene Expression Omnibus (https://www.ncbi.nlm.nih.gov/geo, accessed on 21 August 2026). No laboratory instruments, equipment, or chemical reagents were used in this study. Package and session information are retained with the computational outputs.

The principal analyses comprised periodontal signature discovery, evaluation in the independent RNA-sequencing cohort, and assessment of mapped gene effects, signature scores, and gene-set enrichment in the radiotherapy cohort. Alternative model specifications, influence analyses, and complementary testing procedures assessed sensitivity. Matched random-set comparisons, cellular-proxy analyses, cross-cohort GO comparisons, and clinical associations provided exploratory context. These categories describe the analytical roles within this exploratory study and do not imply prospective preregistration.

Gene-level *p* values were adjusted across all tested genes within each differential-expression comparison. Unless otherwise specified, differential expression required both FDR < 0.05 and |log_2_FC| > 1. These combined criteria were used as a gene-selection rule and do not constitute a formal test that the true effect exceeds the specified fold-change threshold. Score estimates were reported with 95% confidence intervals and two-sided *p* values. Separate multiple-testing families were defined for distinct statistical questions, contrasts, and procedures, as specified in the respective Methods sections and documented in the Supplementary Materials. These families were not assumed to be independent, and correction within them does not provide study-wide error control. Original score-model *p* values were reported as unadjusted, whereas the revised longitudinal component analysis additionally applied Benjamini–Hochberg correction across eight score-by-time comparisons. Unadjusted and adjusted *p* values were distinguished throughout.

Volcano plots displayed log_2_FC against −log_10_ FDR. Heatmaps used gene-wise standardized expression and documented sample ordering. Where color scales were capped for readability, uncapped values were retained for statistical calculations. Forest plots displayed effect estimates and confidence intervals, while GO dot plots encoded enrichment direction, FDR, and significance status. Main and Supplementary Figures were generated from numeric results, with vector PDF and 300-dpi raster exports and accompanying source data.

The revision workflow was organized into sequential scripts operating on the existing processed matrices and analysis objects. Original source scripts, identifier-mapping records, random-set membership, model outputs, and execution logs were retained. Input-file checksums were used to verify that the source data remained unchanged. Automated checks evaluated model reproduction, influence-analysis dimensions, random-set membership, nonoverlapping proxy definitions, GO-result reproduction, and figure completeness.

Cross-cohort correspondence was interpreted as an association between transcriptional patterns. Neither gene-set enrichment nor score similarity was used to infer that periodontitis causally modifies radiation-induced oral injury or establishes clinically useful prediction.

## 5. Conclusions

This exploratory cross-cohort analysis identified partial, analytically dependent transcriptional correspondence between periodontitis and radiotherapy-exposed oral mucosa. The periodontal signature showed strong directional concordance and a significant score association in the independent RNA-sequencing cohort, whereas gene-level replication depended on covariate specification and sample inclusion. In the radiotherapy cohort, neither the mapped replicated periodontal genes nor longitudinal signature scores met their respective significance criteria. Enrichment of the periodontitis-upregulated gene set was significant under the original fixed-correlation CAMERA specification but not when intergene correlation was estimated from the data. Shared GO enrichment highlighted immune recruitment, T-cell regulation, extracellular-matrix organization, and proliferation- and DNA-maintenance-associated processes, providing descriptive evidence of overlap rather than independent confirmation of shared mechanisms. Expression-matched random-set comparisons supported further investigation of this correspondence but did not establish specificity to periodontitis.

These findings should be interpreted in the context of the small longitudinal cohort, unavailable periodontal status in radiotherapy patients, tissue and platform differences, and unresolved contributions from cellular composition. Exploratory clinical analyses did not establish an association with mucositis severity. The results therefore do not demonstrate that periodontitis increases susceptibility to radiation-induced oral mucositis or that the signature has clinical predictive value. Prospective studies integrating periodontal assessment, verified treatment exposure, serial mucosal sampling, concurrent mucositis grading, and cell-resolved profiling are needed to determine the cellular basis, temporal relationships, and clinical relevance of the observed transcriptional overlap.

## Figures and Tables

**Table 1:** Characteristics of the GSE173078 validation cohort and sensitivity analysis of the periodontal signature score. Panel (**A**) participant characteristics. Panel (**B**) association of the signed periodontal signature score with phenotype.

(**A**)				
Phenotype	*n*	Age, years mean (SD)	Female/Male, n (%)	Batch A/B, n (%)
Overall	36	50.0 (15.2)	24 (66.7)/12 (33.3)	18 (50.0)/18 (50.0)
Healthy	12	48.1 (15.6)	10 (83.3)/2 (16.7)	6 (50.0)/6 (50.0)
Gingivitis	12	45.8 (14.2)	8 (66.7)/4 (33.3)	6 (50.0)/6 (50.0)
Periodontitis	12	56.0 (15.1)	6 (50.0)/6 (50.0)	6 (50.0)/6 (50.0)
(**B**)				
Model	Covariate adjustment	Contrast	Adjusted mean difference, β (95% CI)	*p* value
Batch-only sensitivity model	Sequencing batch	Gingivitis vs. healthy	0.71 (−0.52 to 1.94)	0.2475
Batch-only sensitivity model	Sequencing batch	Periodontitis vs. healthy	1.34 (0.12 to 2.57)	0.0329
Fully adjusted primary model	Sequencing batch, age, and sex	Gingivitis vs. healthy	0.66 (−0.55 to 1.86)	0.2748
Fully adjusted primary model	Sequencing batch, age, and sex	Periodontitis vs. healthy	1.66 (0.40 to 2.93)	0.0119

Note: Values in Panel A are presented as mean (SD) or n (%). Female/male and sequencing batch A/B are reported in the indicated order. No age, sex, or batch data were missing. The signed discovery score comprised 97 mapped genes, including 67 upregulated and 30 downregulated genes. Healthy gingiva was the reference phenotype. The batch-only model was adjusted for sequencing batch, whereas the fully adjusted model included sequencing batch, standardized age, and sex. β represents the adjusted mean difference in the signature score. All *p* values are two-sided. CI, confidence interval; SD, standard deviation.

**Table 2:** Candidate marker genes used for the expression-based cellular proxy analyses.

Proxy	Candidate Marker Genes
B cell/plasma cell	CD79A, MS4A1, CD37, CD74, CD19, CD27, MZB1, JCHAIN, TNFRSF17, IGHM, IGKC
Neutrophil	FCGR3B, CSF3R, S100A8, S100A9, S100A12, FPR1, CXCR2
T cell	CD3D, CD3E, CD247, TRAC, IL7R, LCK
Epithelial cell	EPCAM, KRT5, KRT14, KRT16, KRT19, KRT4, KRT13
Fibroblast/extracellular matrix	COL1A1, COL1A2, DCN, LUM, COL3A1, PDGFRA, COL6A1

## Data Availability

The datasets analyzed in this study are publicly available in the National Center for Biotechnology Information Gene Expression Omnibus under accession numbers GSE10334, GSE173078, and GSE103412. The analysis scripts, software dependency records, and execution instructions are publicly available on Zenodo at https://doi.org/10.5281/zenodo.23722502. Complete gene-level results, supporting statistical results (provided as TSV data files), and figure source data are provided in the Supplementary Materials.

## Supplementary Materials

The following supporting information can be downloaded at: https://www.mdpi.com/article/10.3390/ijms27219976/s1.
