# Supplementary material for: Periodontitis-Associated Transcriptional Signatures and Their Correspondence with Oral Mucosal Responses to Radiotherapy: A Cross-Cohort Analysis
Source: Int J Mol Sci. 2026 Nov 7;27(21):9976. doi: 10.3390/ijms27219976 (PMC13764302; doi:10.3390/ijms27219976)
Supplement: Supplementary file 1 [file ijms-27-09976-s001.zip › S Fig.pdf]

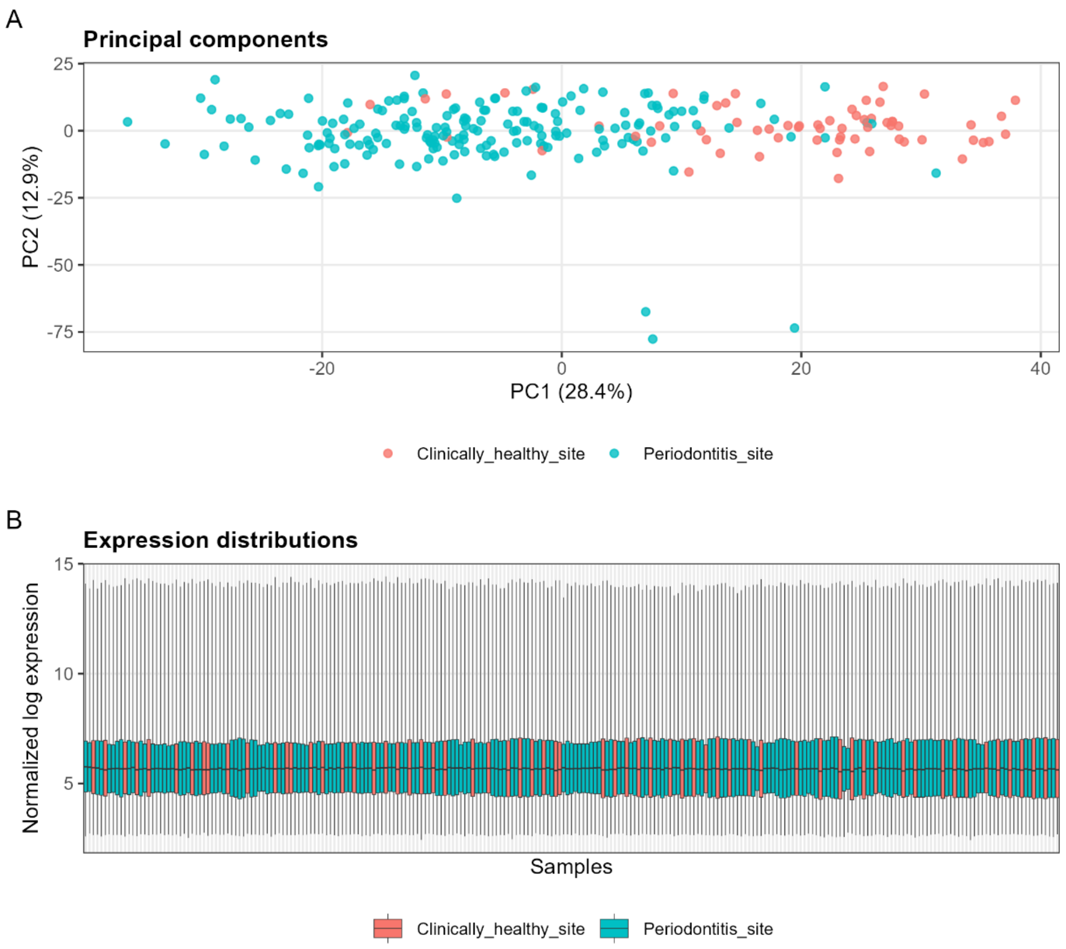

Figure S1. Quality-control assessment of the GSE10334 discovery cohort.

(A) Principal-component analysis (PCA) based on the 2,000 most variable genes in the processed gene-level expression matrix. Each point represents one gingival sample and is colored by clinical site status; percentages indicate the variance explained by each principal component. (B) Distributions of normalized log2 expression values across 247 samples, comprising 64 clinically healthy and 183 periodontitis-affected sites. Boxes indicate the median and interquartile range, and whiskers indicate the minimum and maximum expression values within each sample. The PCA is descriptive; samples were not removed from the primary analysis on the basis of this display.

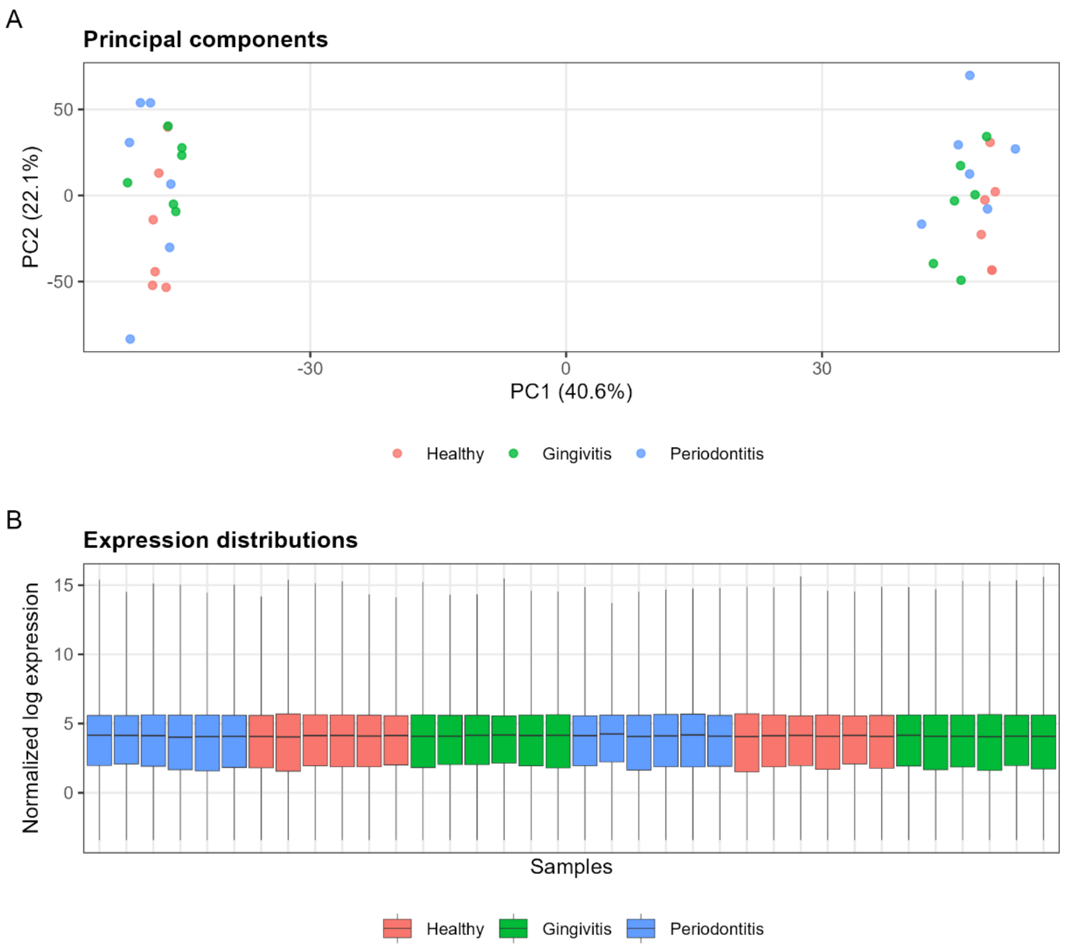

Figure S2. Quality-control assessment of the GSE173078 RNA-sequencing cohort.

(A) PCA based on the 2,000 most variable genes in the processed expression matrix after low-expression filtering. Each point represents one sample and is colored by phenotype; percentages indicate the variance explained by each principal component. (B) Distributions of trimmed mean of M values (TMM)-normalized log counts per million across 36 samples from healthy gingiva, gingivitis, and periodontitis groups (n = 12 per group). Boxes indicate the median and interquartile range, and whiskers indicate the minimum and maximum values within each sample. The plots summarize the processed expression matrix and do not represent covariate-adjusted residuals.

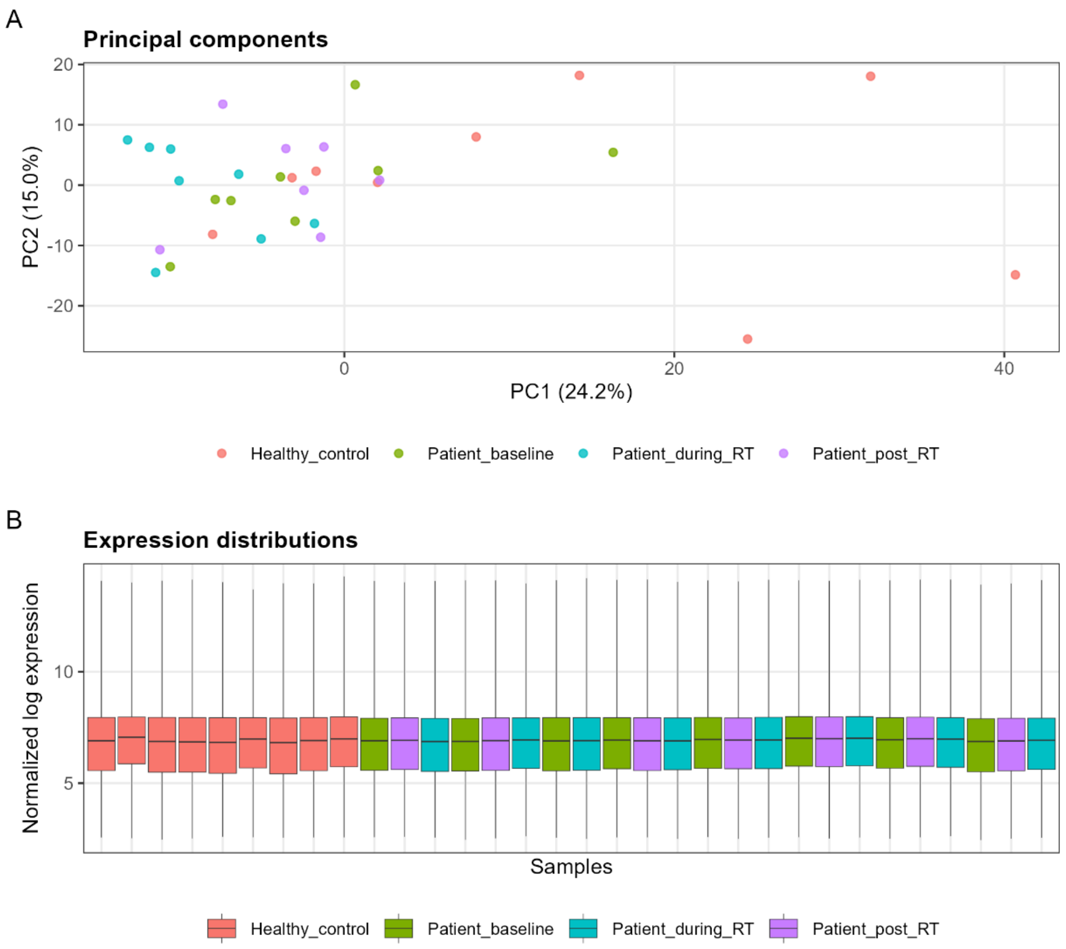

Figure S3. Quality-control assessment of the GSE103412 oral-mucosa cohort.
(A) PCA based on the 2,000 most variable genes in the processed gene-level expression matrix. Each point represents one sample; colors indicate healthy controls, patient baseline, during-radiotherapy, or post-radiotherapy samples, and percentages indicate the variance explained by each principal component. (B) Distributions of normalized log2 expression values across 32 oral-mucosa profiles: nine healthy controls, eight patient baseline samples, eight during-treatment samples, and seven post-treatment samples. Boxes indicate the median and interquartile range, and whiskers indicate the minimum and maximum values within each sample. Healthy controls are included for descriptive quality control; longitudinal patient-effect models use the 23 patient profiles.

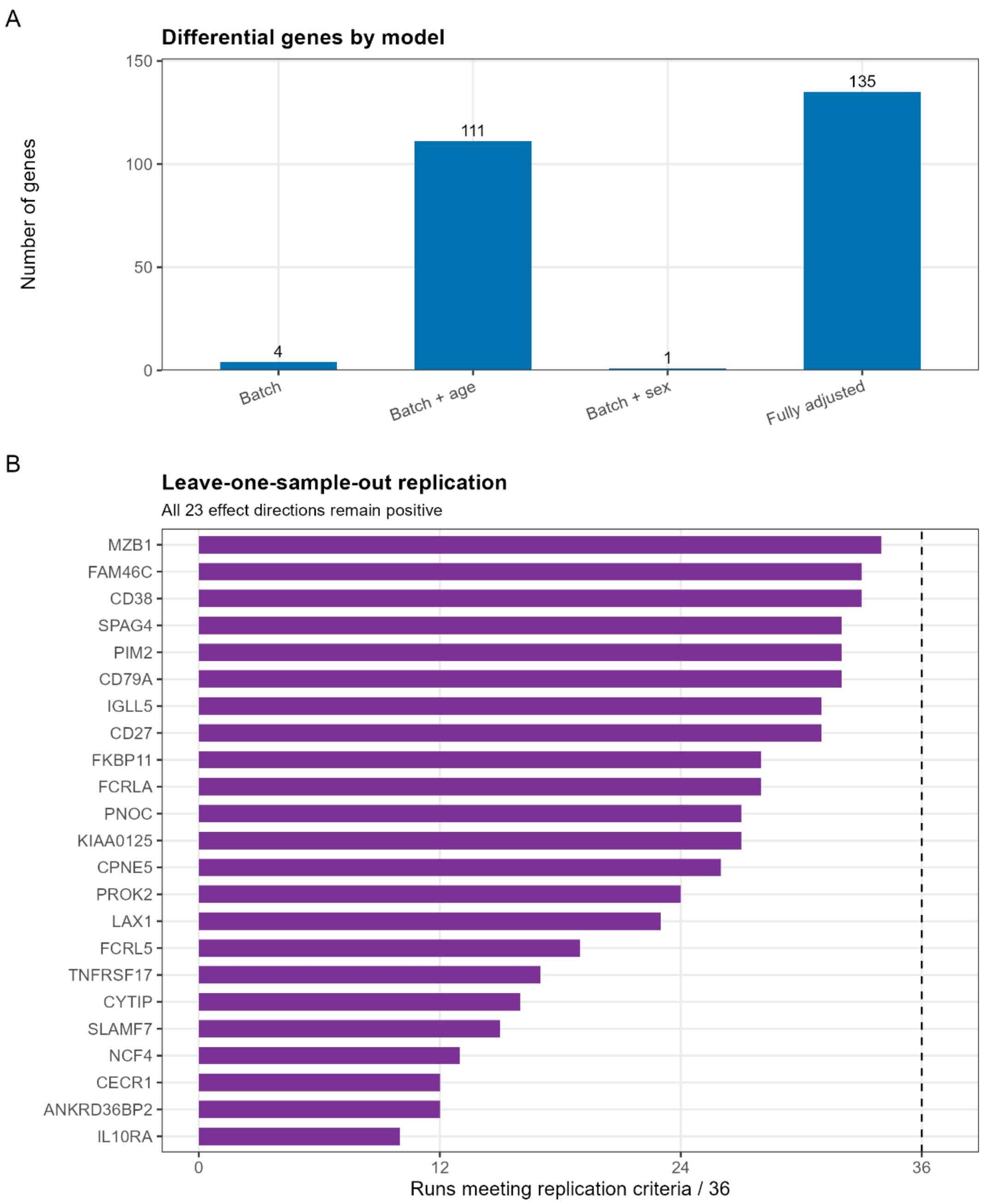

Figure S4. Covariate and sample sensitivity of gene-level replication in GSE173078. (A) Numbers of differentially expressed genes meeting FDR < 0.05 and |log2 fold change| > 1 in models adjusted for batch alone, batch and age, batch and sex, or batch, age, and sex. Values above the bars indicate the respective counts of 4, 111, 1, and 135 genes. (B) Number of leave-one-sample-out analyses in which each of the 23 genes selected by the original fully adjusted model retained concordant direction and met the replication thresholds. Each sample was omitted once, yielding 36 fitted models. The original filtering universe and TMM normalization factors were retained, whereas dispersions and model estimates were refitted. The dashed line denotes all 36 iterations. All 23 genes retained positive effect estimates, but no gene met the replication criteria in every iteration. These analyses assess influence

conditional on the original preprocessing and do not constitute additional independent replication.

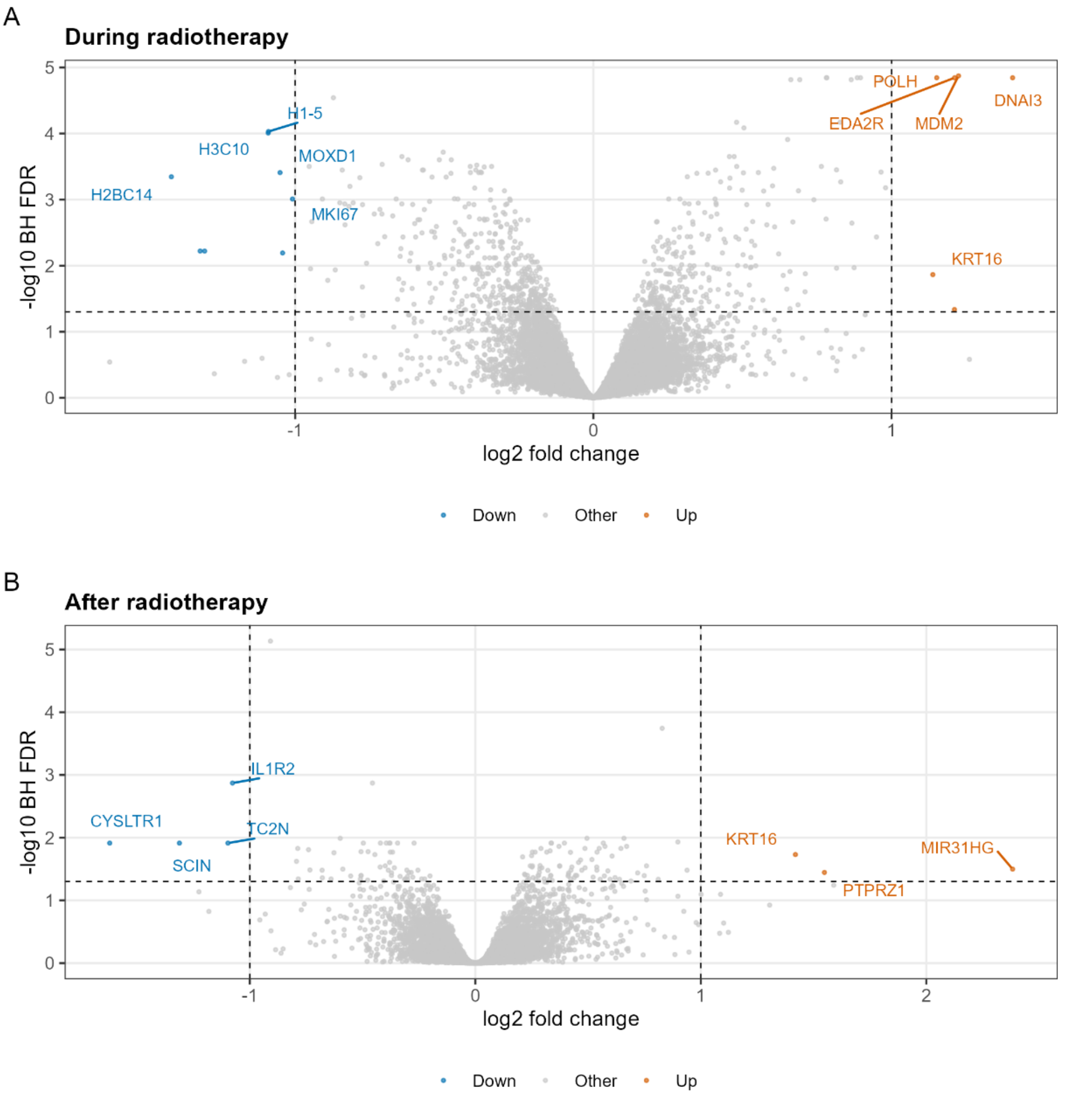

Figure S5. Differential-expression volcano plots for longitudinal radiotherapy comparisons in GSE103412.

Volcano plots comparing samples collected during radiotherapy (A) or after radiotherapy (B) with baseline using models containing fixed patient effects. Orange and blue points denote genes meeting the joint selection rule in the positive and negative directions, respectively, whereas gray points denote genes that did not meet both criteria. Dashed lines mark |log2 fold change| > 1 and Benjamini–Hochberg FDR < 0.05. Fourteen genes, comprising six upregulated and eight downregulated genes, met both criteria during radiotherapy; seven genes, comprising three upregulated and four downregulated genes, met both criteria after radiotherapy. Up to five leading selected genes in each direction are labeled. The analyses used eight baseline/during-treatment pairs and seven baseline/post-treatment pairs within the longitudinal patient-effect model.

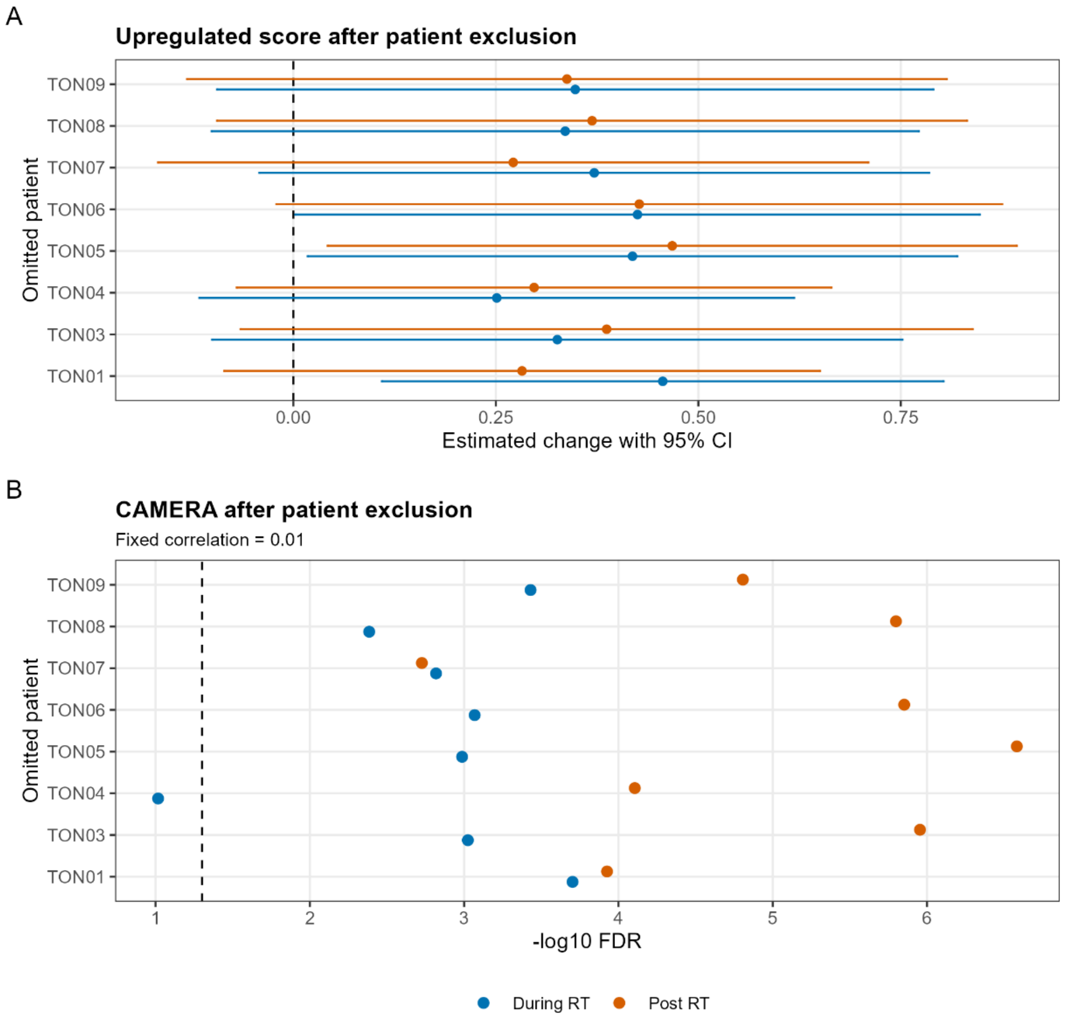

Figure S6. Leave-one-patient-out sensitivity of the periodontitis-upregulated signal in GSE103412.

(A) Estimated changes in the 63-gene upregulated-component score after exclusion of all available samples from each patient in turn. Points indicate model estimates, and horizontal lines indicate 95% CIs; the dashed line marks no change. Gene standardization was held fixed to the original patient-profile matrix. (B) Corresponding CAMERA results for the upregulated gene set under the original fixed intergene correlation of 0.01. Points indicate −log10 FDR, and the dashed line marks FDR = 0.05. Blue and orange denote during-radiotherapy and post-radiotherapy comparisons with baseline, respectively; row labels identify the omitted patient. During-treatment enrichment did not meet FDR < 0.05 in every omission analysis. These results concern the fixed-correlation specification and do not establish robustness to estimated intergene correlation.

A

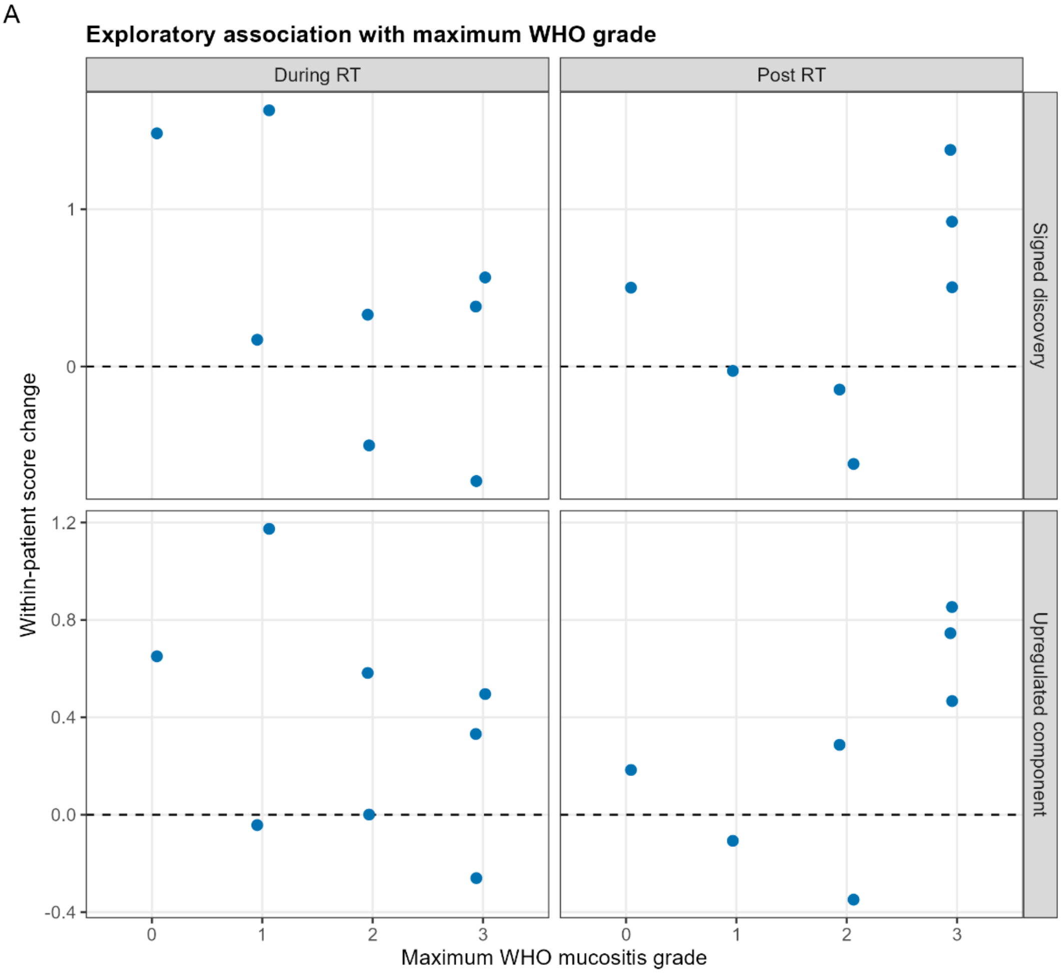

Figure S7. Exploratory associations between periodontal score changes and maximum mucositis severity.

Within-patient changes from baseline in the signed discovery score (upper row) and upregulated-component score (lower row), plotted against patient-level maximum World Health Organization (WHO) mucositis grade. Columns show during-radiotherapy and post-radiotherapy observations. Each point represents one patient, with eight observations during treatment and seven after treatment; horizontal jitter separates overlapping observations. Dashed lines indicate no score change. Maximum WHO grade describes the treatment course and is not a biopsy-specific severity measurement. Associations were assessed using exact two-sided rank-correlation permutation tests, with Benjamini–Hochberg correction across the four score-by-time-point comparisons. None met FDR < 0.05; the analyses are exploratory.

A

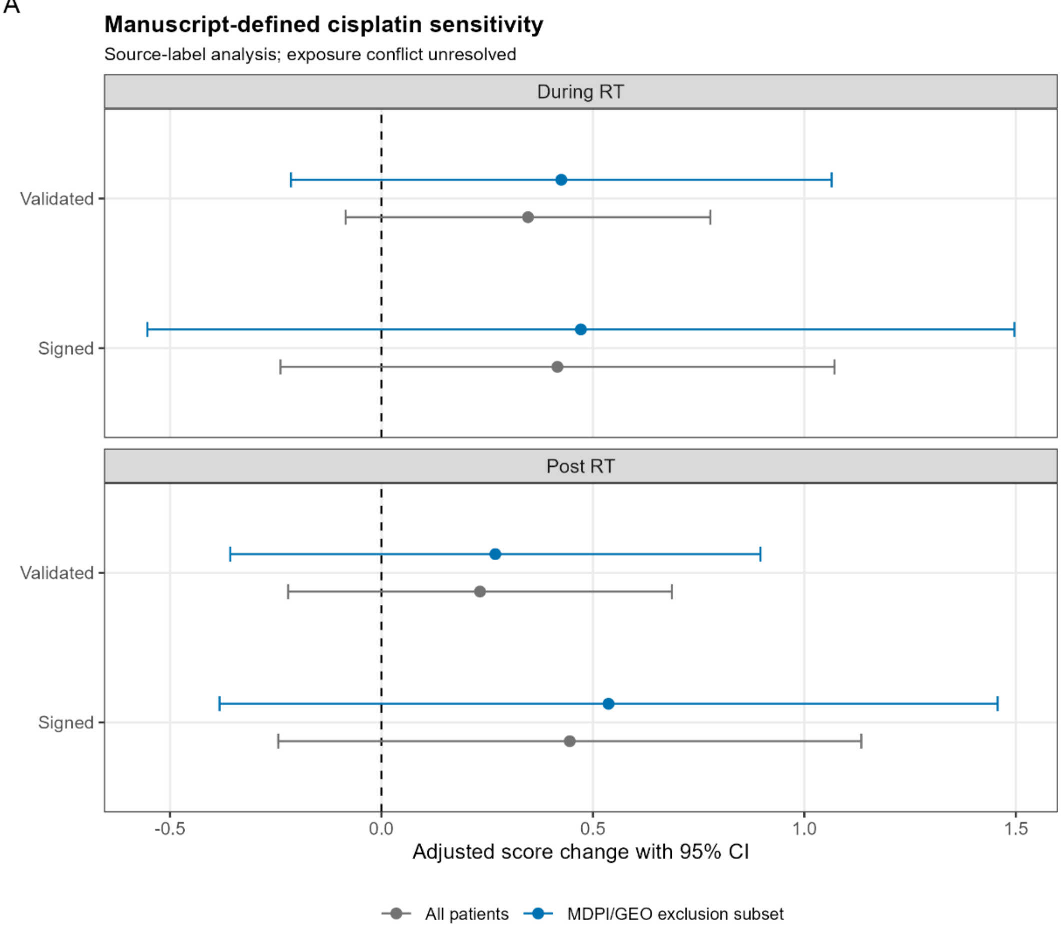

Figure S8. Sensitivity of longitudinal periodontal scores to manuscript-defined cisplatin exclusion.

Adjusted changes in the signed discovery and replicated-gene scores during radiotherapy (upper facet) and after radiotherapy (lower facet), comparing all patients with the exclusion subset defined by the manuscript and deposited GEO treatment labels. Points indicate coefficients from paired score models, and horizontal lines indicate 95% CIs; gray and blue denote all patients and the exclusion subset, respectively. Dashed lines mark no change. The complete cohort provides eight baseline/during-treatment pairs and seven baseline/post-treatment pairs; the exclusion subset provides six and five pairs, respectively. Cisplatin exposure is classified oppositely in the original cohort publication, and both sources are retained in the metadata audit. Accordingly, this figure evaluates sensitivity to source labels and should not be interpreted as a verified radiotherapy-only analysis or a causal comparison of chemotherapy exposure.

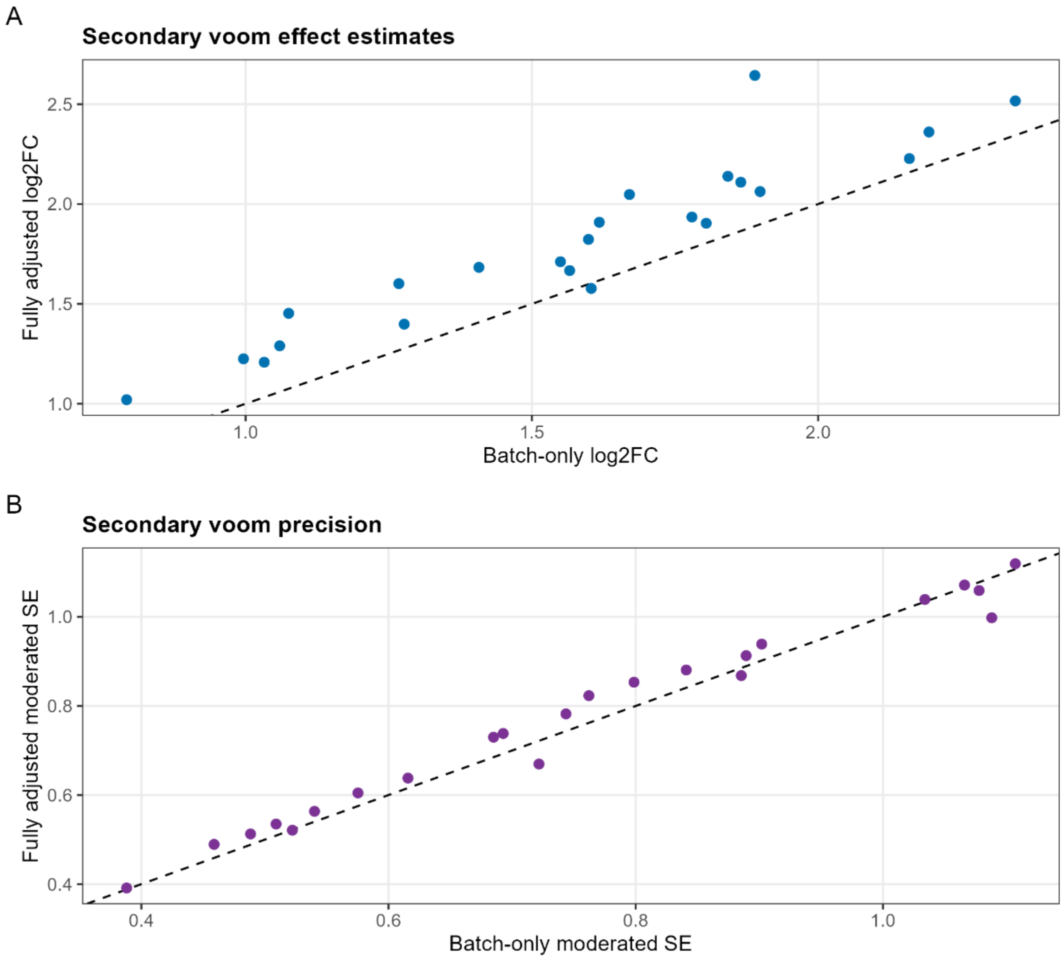

Figure S9. Complementary voom–limma assessment of covariate-dependent effects and precision.

Comparison of log2 fold-change estimates (A) and moderated standard errors (B) between batch-only and fully adjusted voom–limma models for the 23 genes selected by the primary fully adjusted edgeR analysis. Each point represents one gene; the fully adjusted model includes batch, age, and sex. Dashed lines denote equality between model estimates. These standard errors arise from a separately fitted voom–limma sensitivity analysis and are not standard errors from the primary edgeR quasi-likelihood model. Model-specific confidence intervals and the primary edgeR effect, test-statistic, and dispersion outputs are provided in the accompanying tables.
